# Spatial immunophenotypes predict response to anti-PD1 treatment and capture distinct paths of T cell evasion in triple negative breast cancer

**DOI:** 10.1038/s41467-021-25962-0

**Published:** 2021-09-27

**Authors:** Dora Hammerl, John W. M. Martens, Mieke Timmermans, Marcel Smid, Anita M. Trapman-Jansen, Renée Foekens, Olga I. Isaeva, Leonie Voorwerk, Hayri E. Balcioglu, Rebecca Wijers, Iris Nederlof, Roberto Salgado, Hugo Horlings, Marleen Kok, Reno Debets

**Affiliations:** 1 Department of Medical Oncology, Erasmus MC Cancer Institute, Rotterdam, The Netherlands; 2 Division of Tumor Biology & Immunology, The Netherlands Cancer Institute, Amsterdam, The Netherlands; 3 Department of Molecular Oncology & Immunology, The Netherlands Cancer Institute, Amsterdam, The Netherlands; 4 Department of Pathology, GZA-ZNA Ziekenhuizen, Antwerp, Belgium; 5 Division of Research, Peter Mac Callum Cancer Center, Melbourne, Australia; 6 Department of Pathology, The Netherlands Cancer Institute, Amsterdam, The Netherlands; 7 Department of Medical Oncology, The Netherlands Cancer Institute, Amsterdam, The Netherlands

**Keywords:** Cancer, Immunology, Biomarkers, Diseases, Medical research

## Abstract

Only a subgroup of triple-negative breast cancer (TNBC) responds to immune checkpoint inhibitors (ICI). To better understand lack of response to ICI, we analyze 681 TNBCs for spatial immune cell contextures in relation to clinical outcomes and pathways of T cell evasion. Excluded, ignored and inflamed phenotypes can be captured by a gene classifier that predicts prognosis of various cancers as well as anti-PD1 response of metastatic TNBC patients in a phase II trial. The excluded phenotype, which is associated with resistance to anti-PD1, demonstrates deposits of collagen-10, enhanced glycolysis, and activation of TGFβ/VEGF pathways; the ignored phenotype, also associated with resistance to anti-PD1, shows either high density of CD163+ myeloid cells or activation of WNT/PPARγ pathways; whereas the inflamed phenotype, which is associated with response to anti-PD1, revealed necrosis, high density of CLEC9A+ dendritic cells, high TCR clonality independent of neo-antigens, and enhanced expression of T cell co-inhibitory receptors.

## Abstract (web summary)

Only a subset of triple negative breast cancer patients respond to immunotherapy. Here, the authors analysed spatial immune contextures, which can be captured by a gene classifier, in relation to genomic alterations, mechanisms of T cell evasion and response to anti-PD1 treatment.

## Introduction

Triple-negative breast cancer (TNBC) is an aggressive form of breast cancer (BC) (accounting for 10–20% of all BCs) that is characterized by the absence of hormone receptors and has limited therapeutic options. TNBC is considered the most immunogenic BC subtype based on relatively high numbers of tumor-infiltrating lymphocytes (TILs), which is reflected by a higher likelihood of response to immune checkpoint inhibition (ICI) when compared to other BC subtypes^1^. Nevertheless, objective response rates (ORR) to ICI in metastatic TNBC are variable and do not exceed 24% when administered as monotherapy^2^. Clinical benefit has been observed for first-line treatment with the programmed-cell death ligand (PD-L1) blocking antibody (atezolizumab) in combination with nab-paclitaxel, which has been approved by the EMA and FDA for PD-L1-positive metastatic TNBC. Although this combination therapy induces survival benefit in PD-L1-positive TNBC^3^, still a significant proportion of TNBC patients does not benefit from ICI. Moreover, preliminary data of primary TNBC treated with anti-PD1 plus chemotherapy in the neoadjuvant setting suggest that PD-L1 expression is not associated with the benefit of ICI^4,5^. Collectively, these findings point toward the need for better predictive markers and understanding of the underlying immune cell contextures to select TNBC patients for ICI.

Several studies have examined the predictive value of tumor mutational burden (TMB) and TIL abundance in TNBC. While a high TMB has been associated with response to ICI-based therapies in melanoma, lung cancer, and colorectal cancer^6^, no significant association between the TMB and ICI response has been found for TNBC^7–9^. TILs are frequently present in primary TNBC and correlate with good prognosis, as well as response to neoadjuvant chemotherapy and ICI in the metastatic setting^1,8,10–14^. Furthermore, TILs predict overall survival (OS) to anti-PD1 as a monotherapy independent of PD-L1 expression. Emerging evidence now suggests that next to the abundance of TILs, also the cellular composition and activation state of TILs contribute to clinical outcome. For example, the presence of tissue-resident memory CD8+ T cells provides more prognostic information when compared to CD8+ T cells^15^, and hallmarks of an ongoing immune response, such as clonal T cell expansion correlate to anti-PD1 response^8^. In addition, the spatial localization of TILs has prognostic value in TNBC^16,17^. In this regard, three main spatial phenotypes have been identified and recognized for their association with clinical outcome in TNBC, as well as other cancer types^17–19^: inflamed (also reffered to as “hot”; characterized by the presence of intratumoral lymphocytes), excluded (also referred to as “altered”; lymphocytes are restricted to the invasive margin) and ignored (also reffered to as “cold” or “desert”; characterized by lack of lymphocytes). Immune evasive mechanisms, including intrinsic, as well as acquired mechanisms, and their contribution to numbers, cell states, and locations of TILs have been described^20,21^. Such mechanisms include those that inhibit influx and migration of T cells, antigen recognition by T cells or suppression of T cell function^22–28^. Collectively, the above studies describe spatial phenotypes in cancers^17–19^; however, so far it has not been studied whether these phenotypes are predictive of response to ICI and which immune evasive processes underpin these phenotypes in TNBC.

Here, we determined spatial immunophenotypes in four large cohorts of TNBC patients using multiplexed immunofluorescent imaging and next-generation sequencing (NGS). We demonstrated that inflamed, excluded and ignored phenotypes can be accurately assigned by a gene classifier, differentially correlate with prognosis in TNBC and other tumor types, and predict response to anti-PD1 treatment in metastatic TNBC and melanoma. Importantly, spatial immunophenotypes in primary TNBC are characterized by distinct immune determinants, as well as tumor microenvironment (TME) and immune response-mediated paths of T cell evasion. These immunophenotypes provide a rationale to develop therapies specifically for spatial immunophenotypes to enhance response to ICI in TNBC.

## Results

### Spatial contexture of lymphocytes but not myeloid cells is prognostic in TNBC

**Fig. 1:**
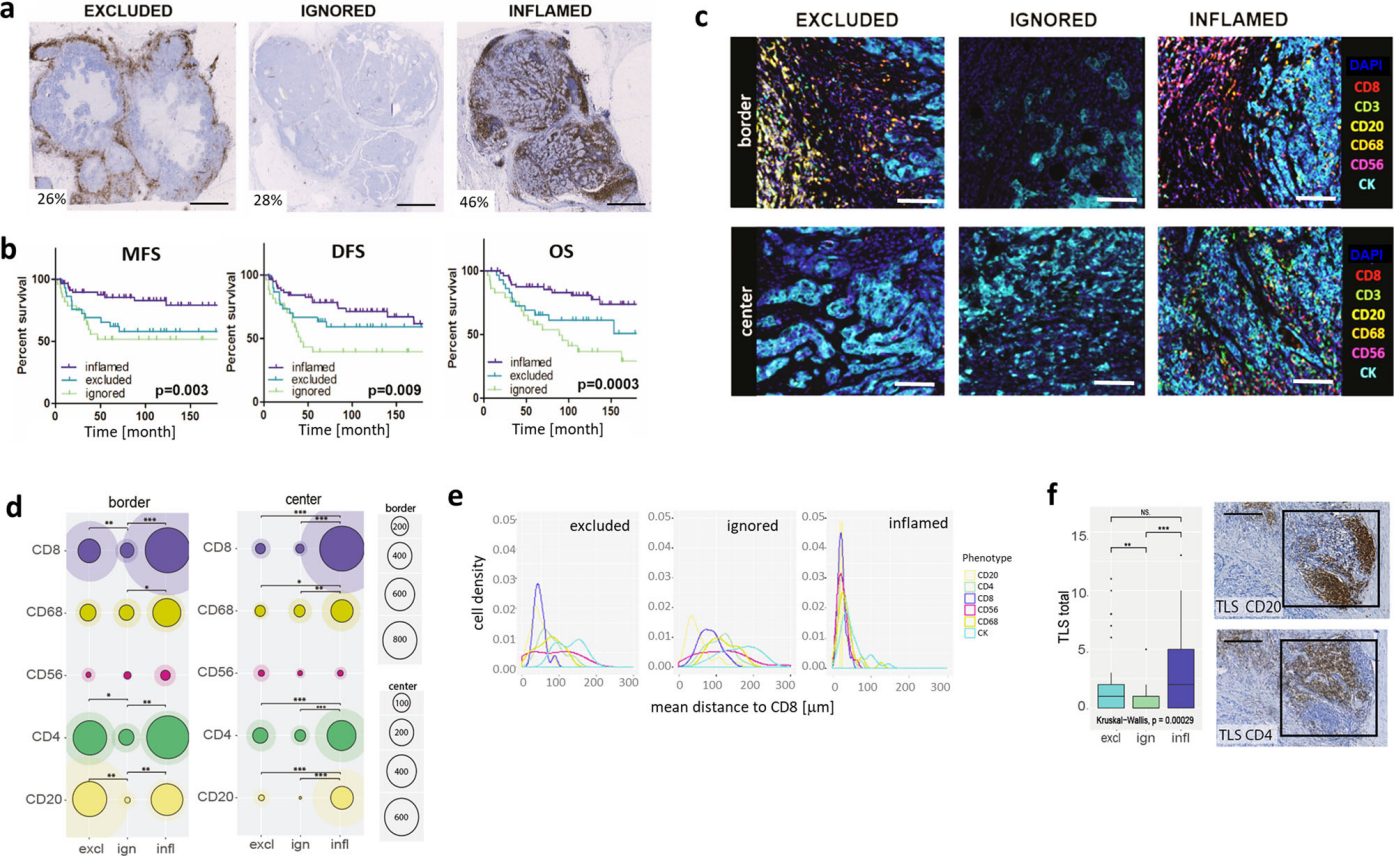
Spatial immune contexture is prognostic in TNBC. **a, b** Representative whole slide images of CD8+ T cell spatial phenotypes with the percentage of patients per phenotype (scalebar corresponds to 5 mm) (A) and corresponding Kaplan–Meier curves for metastasis-free survival (MFS), disease-free survival (DFS), and overall survival (OS) (B, *p* values show two-sided log-rank test; time is displayed in months, *n* = 122 LNN TNBC, of which *n* = 32 excluded, *n* = 27 ignored, and *n* = 61 inflamed). **c** Representative multiplex IF images of immune effector cells at the tumor border and center of each spatial phenotype (CD8+: red, CD3+: green, CD20+:yellow, CD68+: orange, CD56+: magenta, CK:cyan, DAPI: blue; scalebar corresponds to 50 μm). **d** Circle plots show mean and SD of immune cell densities (cells/mm^2^) at border and center for CD8+ (purple), CD68+ (dark yellow), CD56+ (magenta), CD4+ (green), CD20+ (pale yellow), (*n* = 64 LNN TNBC, of which *n* = 18 excluded, *n* = 19 ignored, and *n* = 27 inflamed). **e** Histograms show mean distances in μm between CD8+ T cells and CD8+ (purple), CD68+ (dark yellow), CD56+ (magenta), CD4+ (green), CD20+ (pale yellow) CK+ (cyan) cells (x-axis) versus cell densities (cells/mm^2^, y-axis). **f** Boxplots show median with 25th–75th percentile, range, and outliers displayed as dots of the total number of tertiary lymphoid structures (TLS, identified by consecutive stainings of CD20+ B cells (top) and CD4+ T cells (bottom), see black squares in images, scalebar corresponds to 100 μm; *n* = 134 LNN TNBC, of which *n* = 32 excluded, *n* = 21 ignored, and *n* = 61 inflamed). Significant differences are: ***p < 0.001; **p < 0.01; *p < 0.05, NS, *p* > 0.5 (Kruskal–Wallis, one-sided). Source data are provided within the source data file.

In order to assess tumor-immune interactions in TNBC, CD8+ T cell presence and spatial organization were studied in 236 untreated, primary TNBC using immunohistochemical staining (IHC) of whole slides (Cohort A; for study design see Supplementary Fig. 1 and for clinical details of cohorts see Supplementary Table 1). These patients did not receive adjuvant chemotherapy enabling unbiased testing of the prognostic value of immune markers. We defined three spatial immunophenotypes: excluded (26%; predominant location of CD8+ T cells at tumor border, not center); ignored (28%; negligible presence of CD8+ T cells neither at border nor center) and inflamed (46%; CD8+ T cells evenly distributed across border and center) (see M&M section for detailed criteria of spatial phenotypes) (Fig. 1a, Supplementary Fig. 2a). These spatial phenotypes were significantly associated with survival (distant metastasis-free survival (MFS), disease-free survival (DFS), and overall survival (OS): *p* ≤ 0.009; *n* = 122 lymph-node negative TNBC). Tumors with an inflamed phenotype had the best prognosis (10-year OS: 80%), excluded phenotypes intermediate (10-year OS: 60%, HR:1.45, 95% CI: 0.84–3.3), and ignored phenotypes the worst prognosis (10-year OS: 40%; HR:3, 95% CI: 1.5–5.9) (see Fig. 1b for univariate analysis). Prolonged survival of excluded versus ignored phenotypes was statistically significant for OS, but not MFS nor DFS. Notably, the prognostic value of spatial phenotypes was independent of nodal status, tumor size, or age (see Supplementary Table 2 for multivariable analysis).

In addition to CD8+ T cells, we assessed the presence of other immune effector cells using multiplexed immunofluorescence (IF) imaging of 64 tumors (Fig. 1c, Supplementary Fig. 2b). CD4+ T cells and CD20+ B cells generally co-occurred with CD8+ T cells at the tumor border and center, whereas CD56+ NK cells were hardly present in TNBC (Fig. 1d, Supplementary Fig. 3a–d). For instance, at the tumor border numbers of stromal CD20+ B cells, CD4+ and CD8+ T cells did not differ between excluded and inflamed phenotypes, yet the excluded phenotype had significantly fewer intratumoral B and T cells (Supplementary Fig. 3a, c). Moreover, distances between the CD8+ T cells and tumor cells (CK-positive cells) were significantly larger in excluded versus inflamed phenotypes (Fig. 1e, Supplementary Fig. 3e). Interestingly, despite a lack of lymphocytes in the ignored phenotype, we did observe stromal and intratumoral CD68+ macrophages (Fig. 1d, Supplementary Fig. 3a–d). Notably, densities of stromal CD8+ T cells and intratumoral CD4+, CD8+ T, and CD20+ B cells, but not CD68+ macrophages, demonstrated significant correlations with OS or MFS (Supplementary Fig. 3f). Next, we evaluated the presence of tertiary lymphoid structures (TLS), defined as focal areas that are positive for CD4+ T and CD20+ B cells, which are considered important sites for T cell priming and initiation of an antitumor immune response^29–32^. Interestingly, we observed a high number of TLS at the border of tumors of both the inflamed and excluded phenotype, but not in the ignored phenotype (Fig. 1f). Of note, neither the presence nor abundance of TLS were significantly associated with survival (tested for OS, MFS, and DFS in univariate and multivariable setting), nor when stratified per spatial immunophenotype (Supplementary Table 2).

### A gene classifier of spatial phenotypes predicts outcome to anti-PD1 treatment in TNBC patients

**Fig. 2:**
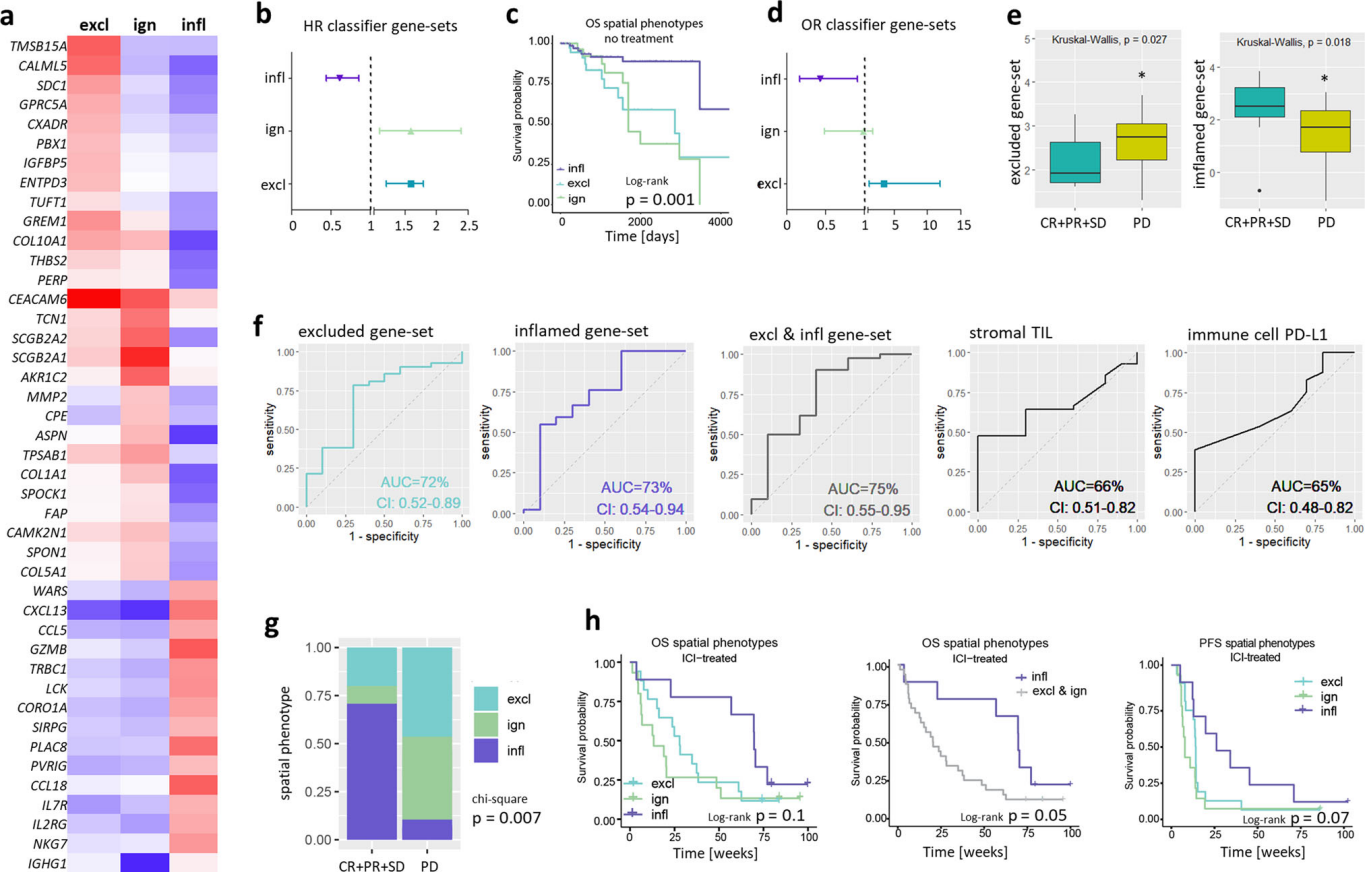
Gene classifier assigns spatial phenotypes of CD8+ T cells and stratifies metastasized TNBC patients according to ICI response. **a** Heatmap showing median expression of classifier genes per spatial phenotype in the discovery set (red: high expression, blue: low expression; Cohort A1, *n* = 101 TNBC). **b** Forestplots showing HRs and CIs (error bars) of classifier gene-sets (Cohort B, *n* = 196 basal-like BC). **c** Kaplan–Meier curves of assigned spatial phenotypes in primary TNBC patients (Cohort E, *n* = 137 TNBC, p-value shows two-sided log-rank test). **d** Forestplots showing Odds Ratios (OR) and CIs (error bars) for response to anti-PD-1 treatment of classifier gene-sets (Cohort D, TONIC trial, *n* = 53 metastatic TNBC). **e** Boxplots displaying the median with 25th–75th percentile and range (outliers are displayed as dots) of the average expression of classifier gene-sets in responding (CR + PR + SD > 24 weeks) and nonresponding (PD) patients (Cohort D, *n* = 53  metastatic TNBC, of which *n* = 10 CR + PR + PD, and *n* = 43 PD). **f** ROC curves predicting clinical response (PR + CR + SD) with areas under the curve (AUC) and CIs for gene sets of excluded-, inflamed- or a combination of the two phenotypes (average expression of respective gene-sets was used) (first three panels), or for standardly used predictive markers, such as frequency of stromal TILs and PDL1 positivity of immune cells (Cohort D) (last two panels). **g** Proportions of assigned spatial phenotypes (excluded: cyan, ignored: green and inflamed: purple) in patients with metastatic TNBC responding or not responding to anti-PD-1 treatment (pretreatment biopsies, *n* = 51) and **h** corresponding survival curves (Cohort D, *p* value shows two-sided log-rank test). Source data are provided within the source data file or can be retrieved under controlled access (see ref. ^8^ for details).

We developed a gene-expression classifier to be able to assess prognostic and predictive values of the spatial immunophenotypes without the need for CD8+ T cell stainings. Briefly, we selected the most discriminative genes (according to differential expression, DE) for the excluded, ignored, and inflamed phenotypes in a discovery set for which both gene expression data and CD8+ T cell stainings were available (Cohort A1, *n* = 101 primary TNBC, Fig. 2a). Using DE and rank-correlations with phenotypes from the discovery set, we assigned spatial phenotypes in an independent validation set (Cohort A2, *n* = 43 primary TNBC; gene expression data and CD8+ stainings), which resulted in correct assignment of spatial phenotypes in 81% of primary TNBC (Table 1; see M&M section for details on classification). Using a second validation set (Cohort F, *n* = 12 metastatic TNBC; gene expression data and CD8+ stainings), we showed the correct assignment of spatial phenotypes in 83% of TN lymph-node metastases (Table 2). Subsequently, the prognostic value of this spatial-phenotype-classifier was tested in an independent cohort of primary, lymph-node negative, systemically untreated BC (Cohort B, *n* = 196 basal-like tumors^10^; only gene expression data available). Genes highly expressed in the excluded or ignored phenotypes included: *THBS2, ASPN, COL10A1, COL5A1 GREM1, SPON1, FAP and SPOCK1*, which were all significantly associated with poor MFS (HR > 1, p < 0.05). On the other hand, genes highly expressed in the inflamed phenotype included: *WARS, CXCL13, CCL5, GZMB, TRBC1, CORO1A, CCL5, CCL18, IL2RG, NKG7, IGHG1*, which were all significantly associated with better MFS (HR < 1, *p* < 0.05) (Supplementary Fig. 4a). Assessment of the entire gene-sets of the excluded and ignored phenotypes were associated with poor prognosis (excluded: HR = 1.8, CI: 1.2–2.7; ignored: HR = 1.6, CI: 1.1–2.4), whereas the gene-set of the inflamed phenotype was associated with good prognosis (HR = 0.62, CI: 0.45–0.86) (Fig. 2b). Upon testing the performance of the gene classifier in a third cohort of primary TNBC patients (Fig. 2c, Cohort E, *n* = 137), we validated the prognostic value of the spatial-phenotype-classifier (log-rank, *p* = 0.001). It is noteworthy that among all BC, basal-like BC had the highest proportion of the inflamed phenotype followed by her2 and luminal-B subtypes (Supplementary Fig. 4b).

**Table 1:** Performance of gene classifier in primary TNBC. Gene classifier (as described in Results section and Fig. 2a) was tested for correct assignment of spatial immunophenotypes in primary TNBC (Cohort A, *n* = 43).

	Spatial immunophenotype (gene-classifier)					
		Excl	Ign	Infl	total	Sensitivity
CD8 stainings	Excl	18	0	0	18	1.0
	Ign	4	5	0	9	0.56
	Infl	3	0	12	15	0.8
	Total	25	5	12	43	
	Specificity	0.72	1.0	1.0

**Table 2:** Performance of gene classifier in LN metastases. Gene classifier (as described in Results section and Fig. 2a) was tested for correct assignment of spatial immunophenotypes in TNBC lymph-node metastases.

	Spatial immunophenotype (gene-classifier)					
		Excl	Ign	Infl	total	Sensitivity
CD8 stainings	Excl	1	1	0	2	0.5
	Ign	0	3	1	4	0.75
	Infl	0	0	6	1	1.0
	Total	1	4	7	12	
	Specificity	1.0	0.75	0.85		

To test the capacity of the spatial-phenotype-classifier to predict outcome after anti-PD1 treatment in TNBC, we applied the classifier to a dataset of metastatic patients from the TONIC trial^8^. In this phase II trial, all patients received anti-PD1 after a short (2 week) immune induction treatment with low dose chemotherapy or irradiation (cohort D, *n* = 53, biopsies from pre- and postinduction treatment metastatic lesions, see Supplementary Table 1 for details). We observed significantly higher frequencies of the excluded (41%) and ignored phenotypes (37%), and decreased frequencies of the inflamed (21%) phenotypes when metastasized TNBC was compared to primary TNBC. This was not dependent on biopsy sites, which supports the prognostic nature of the classifier (Supplementary Fig. 5a, b). Expression of the excluded gene-set was significantly higher in nonresponding (progressive disease (PD) patients) patients (odds-ratio (OR): 3.5; CI: 1.2–11.9), whereas expression of the inflamed gene-set was significantly higher in responding patients (complete response (CR) + partial response (PR) + stable disease (SD) for >24 weeks according to iRECIST criteria^33^) (OR: 0.4; CI: 0.18–0.92) (Fig. 2d, e). No association with therapy response was found for the ignored gene-set (OR = 0.9; CI: 0.5–1.85). When assessing receiver operating characteristic (ROC) as a measure of predictive value of the excluded, inflamed, or combined gene-sets, we observed areas under the curve (AUC) of 0.72 (CI: 0.52–0.89), 0.73 (CI: 0.54–0.94), and 0.75 (CI: 0.55–0.95), respectively. In comparison, PD-L1 expression on immune cells, a biomarker that is currently used in the clinical setting had an AUC of 0.66 (CI: 0.51–0.82) (Fig. 2f). The AUC for sTIL, another marker considered to stratify patients was 0.67 (CI: 0.48–0.82) (Fig. 2f). In addition, nonresponder patients showed enrichment for the excluded and ignored phenotypes (90% of cases), whereas in anti-PD1-responders the inflamed phenotype was enriched (60% of cases, Chi-square, *p* = 0.007, Fig. 2g). In fact, using the spatial-phenotype-classifier we were able to predict outcome after anti-PD1 treatment, i.e., the negative predictive value (NPV) of the inflamed phenotype to therapy response is 0.9, while the positive predictive value (PPV) is 0.6 (Table 3). In line, patients with the excluded and ignored phenotypes had shortened OS when compared to the inflamed phenotype (log-rank, *p* = 0.05, Fig. 2h). Notably, spatial phenotypes predict clinical outcome independent of immune cell PD-L1 but not sTIL (Supplementary Fig. 6).

**Table 3:** Clinical validation of gene classifier. Gene classifier (as described in Results section and Fig. 2a) was tested for prediction of response to anti-PD1 treatment. *PPV* positive predictive value, *NPV* negative predictive value.

	Spatial immunophenotype (gene-classifier)			
		Excl & Ign	Infl	Total
Anti-PD1 treatment	Nonresponder	35	4	39
	Responder	4	6	10
	Total	39	10	49
	NPV	0.9		
	PPV	0.6		

### Prognostic and predictive value of spatial phenotypes in multiple cancers

We applied the spatial-phenotype-classifier to other tumor (sub-)types to assess the prognostic and predictive value in a pan-cancer setting. Spatial phenotypes were significantly prognostic not only in invasive breast cancer BRCA (all subtypes, including ER+) but also in bladder cancer (BLCA), skin cutaneous melanoma (SKCM), cervical squamous cell carcinoma, and endocervical adenocarcinoma (CESC), head and neck squamous cell carcinoma (HNSC) and kidney cancer (KICH), but despite similar trends not in prostate (PRAD), pancreatic (PAAD), lung (LUAD) or colon cancer (COAD) (Cohort E, Supplementary Fig. 4c**)**. Although spatial immunophenotypes have been described for various cancers^22,34^, they have not directly been related to response to ICI treatments. Here we demonstrate that tumors generally responding poorly to ICI, such as PRAD and PAAD, had the highest proportions of the excluded or ignored phenotypes, while tumors generally responding well to ICI, such as SKCM and LUAD, had the highest proportions of the inflamed phenotype (Supplementary Fig. 4b). In line with TNBC, in advanced and metastatic melanoma, where RNAseq data of ICI-treated patients is publicly available^35,36^, we observed that expression of the gene-set of the excluded phenotype was significantly increased in tumors of patients not responding to ICI, and the gene-set of the inflamed phenotype was significantly increased in tumors of patients responding to ICI (Supplementary Fig. 4d). Moreover, the spatial-phenotype-classifier outperformed other, publicly available gene-classifiers that are recognized for capturing lymphocyte activity and location, and for predicting anti-PD1 response in melanoma, such as IFNγ-response, T cell exclusion, and TLS signatures (Supplementary Fig. 7).

### Spatial phenotypes differ in TCR repertoire skewness and mutational signatures but not mutational burden

**Fig. 3:**
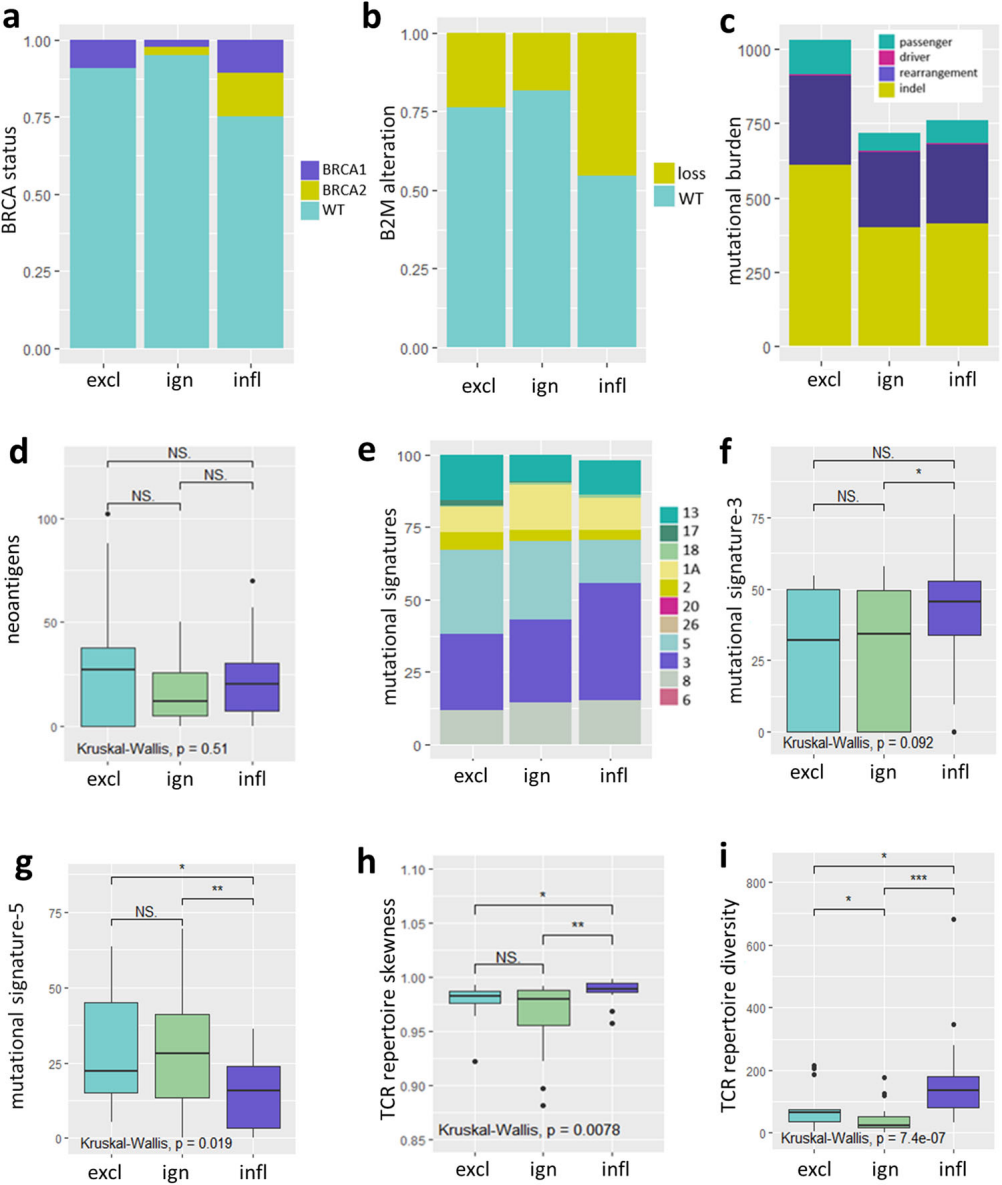
Genomic features of spatial phenotypes. The following parameters were tested for differential presence in spatial phenotypes (determined by the gene-classifier) in TNBC: **a** BRCA status (proportion, BRCA1: purple, BRCA2: yellow, WT: cyan). **b** Loss of β2-microglobin (copy number, B2M-loss: yellow, B2M-wt: cyan). **c** Total number of different types of mutations (passenger mutations: cyan, driver mutations: magenta, structural rearrangements: purple, indels: yellow). **d** Total number of predicted neo-antigens. **e** Proportions of most abundant mutational signatures. **f**, **g** Frequencies of signatures-3 and 5. **h** TCR repertoire skewness (based on the gini-simpson index). **i** Total number of different TCR-Vβ reads. For all above parameters Cohort C (*n* = 66 TNBC, comprising *n* = 13 excluded, *n* = 29 ignored, and *n* = 24 inflamed) was used, spatial phenotypes were assigned according to the classifier. All boxplots display the median with 25th–75th percentile, range, and outliers are displayed as dots. Significant differences are: ***p < 0.001; **p < 0.01; *p < 0.05, NS, *p* > 0.5 (Kruskal–Wallis, one-sided). Source data are provided within the source data file.

In order to test for potential drivers of spatial phenotypes, we first studied clinicopathological and genomic features in lymph-node negative, systemically untreated, primary TNBC (Cohorts A and C). Spatial phenotypes were not associated with mitotic activity index (MAI), tumor grade, tumor stage, or histological subtypes, except for tumors with medullary features that were (as expected) solely comprised of the inflamed phenotype (Supplementary Fig. 8). In addition, following assignment of spatial phenotypes to Cohort C (*n* = 66; RNAseq and WGS data), we observed that spatial phenotypes did neither differ with respect to frequency of *BRCA1* or *BRCA2* germline mutations (Fig. 3a), frequency of β2 Microglobulin loss (Fig. 3b) nor TMB or types of genomic alterations, including nonsynonymous SNV (passenger and driver mutations combined), exonic frameshifts, indels (Fig. 3c) or predicted neo-antigens (Fig. 3d). In contrast, spatial phenotypes did differ with respect to mutational signatures and TCR clonality (Fig. 3e–i). For instance, mutational signature-3 (related to homologous recombination deficiency) was enriched in the inflamed phenotype and signature-5 (related to age) was significantly enriched in the ignored and excluded phenotypes (Fig. 3f, g). The highest TCR-Vβ diversity, as well as the most skewed TCR-Vβ repertoire (harboring clonally expanded reads) were observed in the inflamed phenotype, and both these parameters were equally low in the excluded and ignored phenotypes (Fig. 3h, i).

### Spatial phenotypes are characterized by distinct immune evasive pathways

**Fig. 4:**
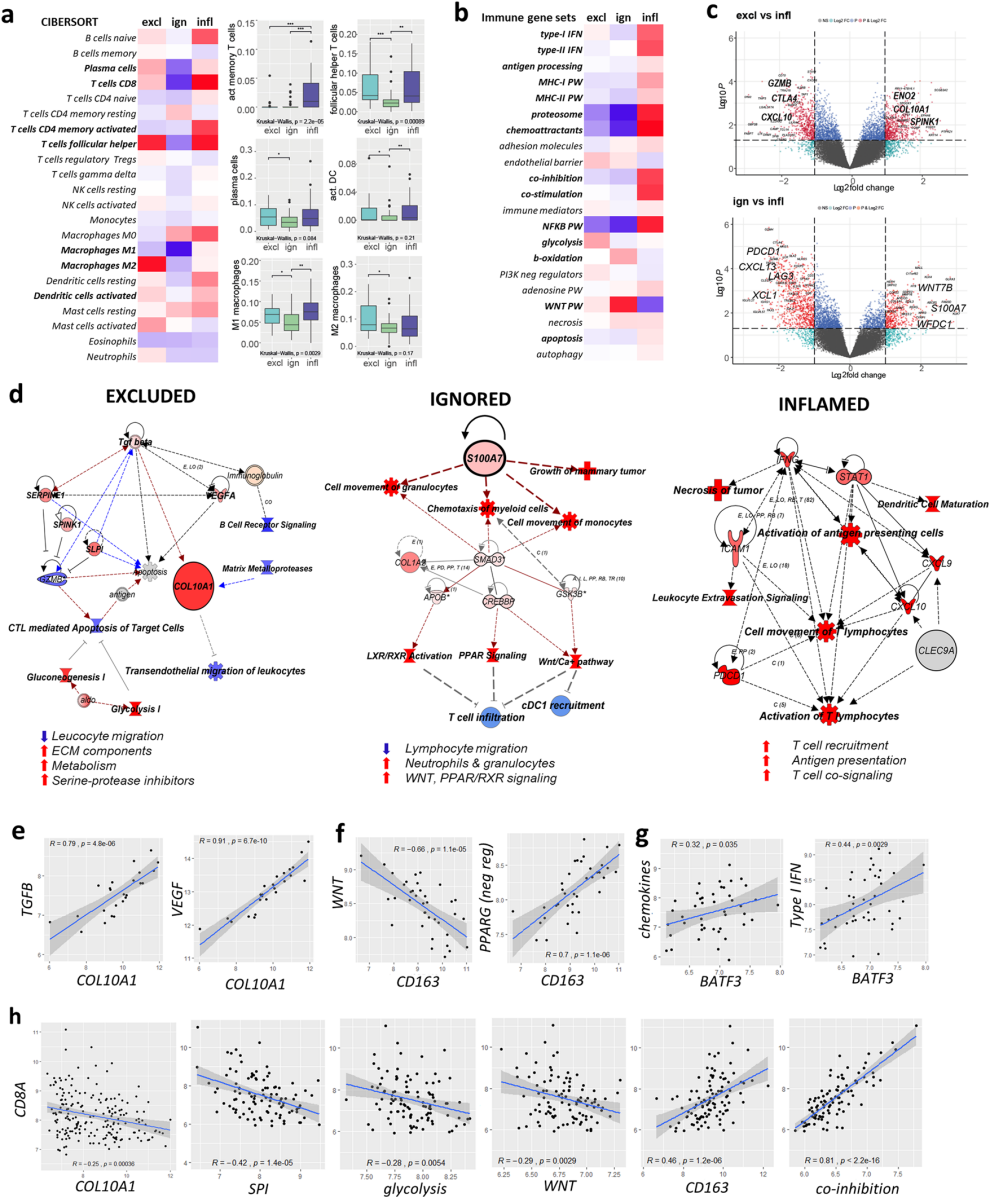
Spatial phenotypes interrogated for immune determinants and evasive pathways. **a** Heatmap showing scaled average frequencies of immune cells based on Cibersort deconvolution (red: high, blue: low, immune cell subsets with significant differences among spatial phenotypes are indicated in bold); corresponding boxplots show median with 25th–75th percentile and range (outliers are displayed as dots) of immune cell subsets with differential abundances among spatial phenotypes (*n* = 101 LNN TNBC, of which *n* = 24 excluded, *n* = 33 ignored, and *n* = 44 inflamed). **b** Heatmap showing scaled average expression of gene-sets related to T cell evasion (differential gene-sets are indicated in bold). **c** Volcano plot of differential gene expressions between excluded and inflamed (upper), and ignored and inflamed phenotypes (lower); top DE genes related to T cell evasion are shown in bold. **d** IPA analyses of cells, molecules, and pathways associated with spatial phenotypes (red: upregulated, blue:downregulated); and lists of major characteristics per spatial phenotype (bottom). **e** Correlations between expressions of *COL10A1* and *TGFB*- or *VEGF*-signaling in the excluded phenotype. **f** Correlations between expressions of *CD163* and *WNT* targets or negative regulators of *PPAR* genes in the ignored phenotype. **g** Correlations between the presence of activated dendritic cells (according to *BATF3* expression) and expressions of *chemokines* or *type-I IFN* genes in the inflamed phenotype. **h** Correlations between expressions of *CD8A* and various T cell evasive genes/gene-sets (all phenotypes) (all correlations show regression coefiicients and *p* values). Significant differences are: ***p < 0.001; **p < 0.01; *p < 0.05, NS, *p* > 0.05 (Kruskal–Wallis, one-sided). Source data are provided as the source data file.

**Fig. 5:**
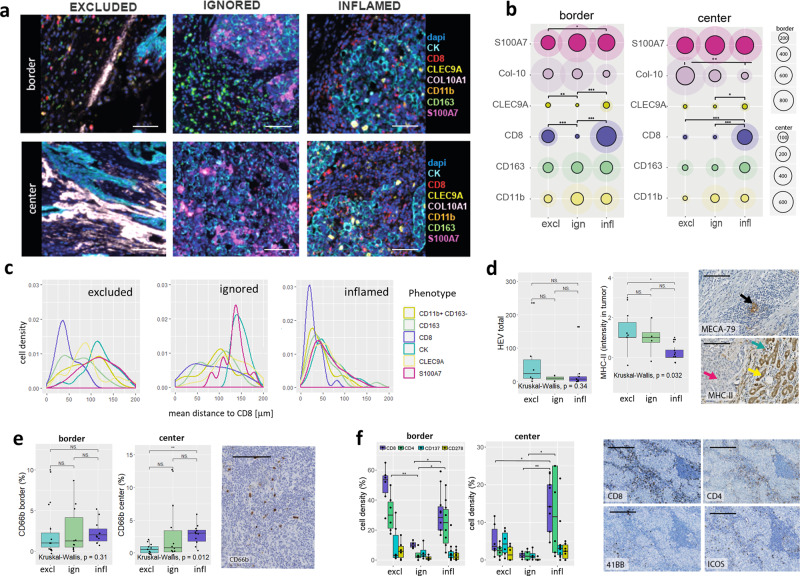
Spatial immunophenotypes are characterized by distinct T cell evasive mechanisms. **a** Representative images of cells and molecules related to spatial phenotypes (spatial phenotype panel) at the tumor border and center (CD11b (orange), CD163 (green), CD8 (red), CK (cyan), CLEC9A (yellow), or S100A7 (magenta); scalebar corresponds to 50 μm). **b** Circle plots show mean and SD of cell densities at border and center regions per mm^2^ for CD11b (orange), CD163 (green), CD8 (purple), CK (cyan), CLEC9A (yellow), or S100A7 (magenta); Collagen-10 (pink) is displayed as positive tissue area in μm^2^/100 for visualization purpose (*n* = 68 TNBC comprising *n* = 20 excluded, *n* = 22 ignored, and *n* = 26 inflamed). **c** Histograms show mean distances in μm between CD8+ T cells and CD11b (orange), CD163 (green), CD8 (purple), CK (cyan), CLEC9A (yellow), or S100A7 (magenta) (x-axis) versus cell densities (y-axis) (*n* = 68 TNBC of which *n* = 20 excluded, *n* = 22 ignored, and *n* = 26 inflamed). **d** Boxplots show median with 25th–75th percentile and range (outliers are displayed as dots) of numbers of high endothelial venules (HEV, identified via MECA-79 staining, black arrow) and MHC-II expression of tumor cells for excluded (cyan), ignored (green), and inflamed (purple) TNBC (no distinction between border and center, pink arrow: tumor cells; yellow arrow: adjacent normal breast lobules; green arrow: immune cells, scalebar corresponds to 100 μm, *n* = 20 TNBC, of which *n* = 6 excluded, *n* = 4 ignored, and *n* = 10 inflamed). **e** Boxplots show median with 25th–75th percentile and range (outliers are displayed as dots) of neutrophil densities (CD66b+) at border and center for excluded (cyan), ignored (green), and inflamed (purple) TNBC and the representative image is shown, scalebar corresponds to 100 μm, (*n* = 32 TNBC, of which =11 excluded, *n* = 10 ignored, and *n* = 11 inflamed). **f** Boxplots show median with 25th–75th percentile and range (outliers are displayed as dots) of numbers of different T cell markers stained on consecutive slides, and representative images (CD8 (purple), CD4 (green), 41BB (cyan), and ICOS (yellow); scalebar corresponds to 100 μm, *n* = 20 TNBC, of which *n* = 6 excluded, *n* = 4 ignored, and *n* = 10 inflamed). Significant differences are: ***p < 0.001; **p < 0.01; *p < 0.05, NS, *p* > 0.05 (Kruskal–Wallis, one-sided). Source data are provided within source data file.

Next, we studied whether spatial phenotypes capture different modes of immune-evasion (Cohort A, Figs. 4 and 5). Immune cell deconvolution by Cibersort^37^ confirmed the above observations (Fig. 1d) with respect to the abundance of immune effector cells and particularly revealed differential frequencies of plasma cells, activated memory T cells, follicular helper T cells, activated dendritic cells (DC), and M1- and M2 macrophages (Fig. 4a). Subsequently, we evaluated the expression of gene-sets related to various mechanisms of T cell evasion^10^, complemented with Ingenuity Pathway Analysis (IPA®) and verified with gene-set enrichment analysis. Using this approach, we observed that the excluded phenotype was characterized by enhanced expression of genes associated with endothelial barrier, glycolysis, serine protease inhibition (SPI), and extracellular matrix (ECM) remodeling (Fig. 4b–d, see Fig. 4c for examples of individual genes, Supplementary Fig. 9a, Supplementary Fig. 10); notably all these pathways were inter-linked with the *TGFβ* pathway (Fig. 4d, 4e, Supplementary Fig. 9a, b, Supplementary Fig. 10). One of the most upregulated genes in the excluded phenotype (compared to the inflamed phenotype) was *COL10A1* (Fig. 4c), the expression of which was strongly correlated to the *TGFB-* and *VEGF*- signaling pathways while being inversely correlated to the expression of *CD8A* (Fig. 4e, h). The ignored phenotype was characterized by increased expression of genes associated with β-oxidation (Fig. 4b) as well as the *WNT*, *PPAR*, *LXR/RXR*, and *MAPK* pathways (Fig. 4c, d, Supplementary Fig. 10). Moreover, the ignored phenotype showed enhanced gene expression of *S100A7* (Fig. 4c), a molecule that has been reported to promote oncogenesis and act as a chemo-attractant for M2 macrophages and other suppressive myeloid cells^38^. Of the above oncogenic pathways in particular *WNT* was inversely correlated with the expression of *CD8A* as well as *CD163* (Fig. 4f, h Supplementary Fig. 9b). Last, the inflamed phenotype showed enhanced expression of genes associated with necrosis, TNF-signaling, type-I and type-II IFN, antigen processing and presentation, T cell co-stimulation, but also co-inhibition (Fig. 4b, c, Supplementary Fig. 10a, b), which were all inter-related (Supplementary Fig. 9b). Importantly, the inflamed phenotype showed high gene expression of the T cell chemo-attractants *CXCL9* and *CXCL10* (Fig. 4c), which according to our immune cell deconvolution and pathway analyses are derived from activated (BATF3/CLEC9A-positive) conventional DC (cDC1, Fig. 4g). *CD163* and T cell co-inhibition^27^, generally down-stream of an immune response, were correlated with the expression of *CD8A* (Fig. 4h).

Multiplex IF demonstrated that collagen-10 was deposited into stromal areas between the tumor and immune cells at the tumor center in the excluded phenotype, (Fig. 5a, b). To assess how the entry of T cells may be affected by such a physical barrier, we evaluated the presence of high endothelial venules (HEV, identified via MECA-79 stainings), and observed that these were present at high numbers at the border, as well as the center of excluded phenotypes (Fig. 5d). In the ignored phenotype, IF showed (albeit only in subset of ignored tumors) that very high S100A7 expression by tumor cells (highest of all spatial phenotypes) was accompanied by high frequencies of CD163+ macrophages (Fig. 5a, middle panel, b). Even though tumor-associated macrophages were not unique for the ignored phenotype, and were present at particularly high densities in the center of inflamed and to a lesser extent in excluded phenotypes, nearest-neighbor analysis revealed that macrophages and myeloid cells showed relatively low distances to CD8+ T cells, regardless of frequencies and spatial phenotypes (Fig. 5c). CD66b+ neutrophils (another immune cell type that has been reported for its immune-suppressive effects in the TME^39^) co-occurred with macrophages and myeloid cells and were found to be present at high numbers in the same subset of the ignored phenotype (Fig. 5e). Notably, the ignored phenotypes that did not show high M2 and neutrophil densities were characterized by enhanced expression of *WNT* targets. In the inflamed phenotype, IF revealed significantly enhanced numbers of stromal, as well as intratumoral CLEC9A+ DC (Fig. 5a, b). Interestingly, and despite overall low abundances of these cells (regardless of spatial phenotype), CLEC9A+ DC were found in relatively close proximity to CD8+ T cells (Fig. 5c), and their cell densities significantly correlated with those of CD8+ T cells (Supplementary Fig. 9c), pointing to the recognized immune-enhancing action governed by cDC1 cells^40^. Nevertheless, and despite high densities of T cells and TLS, only a small fraction of CD4+ and CD8+ T cells in the inflamed phenotype expressed the co-stimulatory receptors ICOS or 41BB, which co-ocurred with a significantly decreased MHC-II expression by tumor cells (Fig. 5d, f).

## Discussion

In this study, using cohorts of in total 681 patients with TNBC, 2706 with other types of BC, and 4003 with other cancers, we have analyzed spatial immunophenotypes in relation to prognosis and response to anti-PD1 treatment, as well as genomic features and T cell evasion. Our results, present that spatial immunophenotypes predict response to ICI in TNBC, and are characterized by distinct T cell evasive pathways that provide a rationale to develop spatial phenotype-specific therapies for ICI-refractory TNBC.

We have developed and validated a spatial-phenotype-classifier that accurately predicts spatial localization of CD8+ T cells in primary as well as metastasized TNBC. Next to its prognostic value in TNBC, this classifier has prognostic value in various tumor types (BC, CESC, HNSC, KICH, BLCA, SKCM), which is in line with recent reports^17,18,41^, and suggests that the classifier may be applied to different histologies. Strikingly, we found that this classifier predicts resistance to anti-PD1 treatment in metastatic TNBC, as well as melanoma. In case of TNBC, we report an NPV as high as 0.9, which is not achieved with the currently used predictor PD-L1. In fact, the spatial immuophenotype classifier acts independently of PD-L1 (Supplementary Fig. 6c) and outperforms alternative classifiers that relate to lymphocyte activity and location (Supplementary Fig 7). Notably, TLS, whether captured by staining (as performed in this study) or a gene signature, was neither significantly associated with survival nor anti-PD1 response, irrespective of stratification per spatial immunophenotype (Supplementary Table 2, Supplementary Fig. 7), indicating that further research is needed to determine the exact role of TLS in shaping antitumor immune responses in TNBC.

Although not excluding CD8 stainings, our observations imply that gene-based classification of spatial immunophenotypes would enable early identification of non-responders and facilitate decision-making by clinical oncologists with respect to the treatment of TNBC patients with ICI. Improved decision-making would prevent non-responding patients to receive ineffective and expensive treatment, and potentially challenge the diagnostic need for tissue stainings, which require whole tissue sections that are often not available, as well as uniform staining protocols and training of pathologists. Regarding the diagnostic implementation of the gene classifier, expressions from those genes that are part of our gene classifier could be developed into a routine tool. Alternatively, NGS-techniques, expected to become part of systemic evaluations of tumor tissues for targetable alterations in the near future at departments of Pathology of Medical Centers, could be used towards the application of the gene classifier.

**Fig. 6:**
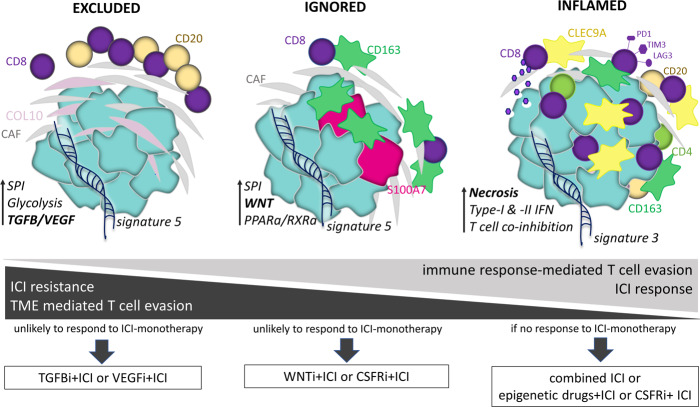
Illustration of immune contextures per spatial phenotype in relation to paths of T cell evasion as well as response to ICI. Distinctive and dominant pathways (in bold), when targeted in an immunophenotype-specific manner (in boxes), would sensitize TNBC to ICI (see Discussion section for details).

The excluded and ignored phenotypes do not respond to anti-PD1 and can be considered variants of cold tumors. In addition to the predictive value of spatial phenotypes, in the TONIC trial, we observed that proportions of the inflamed phenotype increase following induction treatment with cisplatin and doxorubicin (Supplementary Fig. 5c), suggesting that spatial phenotypes show plasticity and that cold TNBC (ie. excluded and ignored) can be primed for treatment with ICI. In this regard, the distinct paths of T cell evasion that characterize these phenotypes provide actionable targets in order to prime for treatment with ICI (illustrated in Fig. 6, and discussed below). In case of the excluded phenotype, we argue that inhibitors of TGFβ, such as the bifunctional anti-PDL-1 mAb/TGFβ trap M7824, and inhibitors of VEGF receptor kinases, such as cediranib, both being in clinical development for TNBC^42,43^ and the latter being FDA-approved for other malignancies^44,45^, can potentially prime for ICI. In case of the ignored phenotype, blockers of the WNT pathway, such as WNT974 and/or drugs that target M2 macrophages, such as pexidartinib, a CSF1R inhibitor that depletes M2 macrophages, are of interest, and are currently being tested in TNBC^46^. The inflamed phenotype, being enriched in patients responding to anti-PD1 treatment, would be the phenotype of choice to start combination ICI treatment. In case ICIs are not effective, this phenotype could potentially benefit from combining multiple ICIs or priming with CSF1R inhibitors that target M2 macrophages. Another mode of priming the inflamed phenotype could be reactivation of type I/II IFN pathways, thereby re-boosting antigen presentation, as well as recruitment and function of intratumoral CD8 T cells^39^; to this end, an option could be the epigenetic drug decitabine that is approved for other indications and has shown promising results in preclinical studies of TNBC^47^.

The above-mentioned targets are part of larger immune networks that were revealed upon integrative analyses of TNBC samples using NGS and multiplexed IF. The charting of these larger networks enabled the identification of TME- and immune response-mediated paths of T cell evasion and their relationship to ICI response. Following this approach, we observed that the excluded phenotype was characterized by CD4+, CD8+, CD20+, and CD56+ lymphocytes that were preferentially located at the tumor border at large distances from tumor cells. This phenotype had high expression of collagen-10, which is not present in normal tissues^48^, is associated with epithelial-to-mesenchymal transition^49^, as well as poor survival in TNBC and various other tumor types^50^. Recently, it has been suggested, based on collagen fiber density (not further specified) and in silico modeling of T cell influx, that T cell exclusion in TNBC is regulated by chemorepellents rather than barriers of extracellular matrix^51^. In contrast, our gene expression and in situ stainings (Figs. 4c, h and 5a, b) strongly suggest that T cell exclusion is due to collagen-10 deposition, possibly hinting toward a unique role of collagen-10 imposing a physical barrier to T cell influx. Next to the collagen barrier, our data point to enhanced tumor cell glycolysis, which has been reported to suppress T cell-mediated apoptosis of TNBC in vitro^52^, and which may further promote T cell exclusion. In addition, several serpins and other protease inhibitors, such as *SERPINE1*, *SPINK1*, and *SLPI*, demonstrated high gene expression. These enzyme inhibitors limit the activity of matrix metalloproteases or granzymes, thereby again potentially inhibiting T cell influx or T cell-mediated apoptosis of tumor cells^53^. All immune evasive pathways associated with the excluded phenotype are inter-related (Supplementary Fig. 9b) and strongly correlate to the expression of *TGFB* and *VEGF* pathways (Fig. 4e), which most likely represent upstream regulators that contribute to TME-mediated T cell evasion. Interestingly, a study by Mariathasan and colleagues showed that T cell exclusion in bladder cancer patients was attributed to stromal remodeling via TGFβ and revealed in a mammary mouse model that pharmacological blockade of TGFβ promotes T cell inflammation^54^.

The ignored phenotype was characterized by no or very low densities of CD8+ T cells and either showed high expression of target genes of the *WNT* and *PPARG/RXR* pathways or contained CD163+ macrophages and CD66b+ neutrophils. Activation of the *WNT* pathway promotes T cell exclusion in bladder cancer^24^ and melanoma, and in the latter, the mechanism has been attributed to the failure of cDC1 recruitment^26^. In line with these data, we found inverse correlations between *WNT* pathway activity and the presence of CLEC9A+ DC and CD8+ T cells, as well as TCR repertoire skewness. Also activation of the *PPARG/RXR* pathway has been related to T cell exclusion and resistance to ICI in bladder cancer^55^, suggesting that the occurrence of both *WNT* and *PPAR* pathways are representative of pan-cancer mechanisms of TME-mediated T cell evasion. Notably, we observed strong inverse correlations with either pathway and the abundance of CD163+ cells (Fig. 4f), and argue that the presence of M2 macrophages represents a second immune escape mechanism of the ignored phenotype. Murine models of BC revealed that S100A7 expression induced M2 macrophage recruitment and promoted metastasis^38^. In the current study with patient materials, however, we found that numbers of S100A7+ tumor cells, as well as CD163+ cells, located at the border, were positively correlated with MFS (Supplementary Fig. 11) and (low) frequencies of CD8+ T cells, arguing that recruitment of these myeloid cells is part of a negative-feedback loop that follows an initial immune response.

Finally, the inflamed phenotype was characterized by high numbers of intratumoral CLEC9A+ DC and lymphocytes. The prognostic value of TILs was mainly attributed to T and B cells located in tumor regions, a finding that is in line with earlier observations showing that proximity to tumor cells is a pre-requisite for the effective anti-tumor activity of lymphocytes^41^. The inflamed phenotype had a high TCR clonality independent of the level of neo-antigens and showed the highest expression of genes associated with immunogenic cell death, type I/II IFNs, and chemo-attractants. Interestingly, we observed that gene sets associated with necrosis, but not any other form of cell death, strongly correlated with densities of CD8+ T cells (Supplementary Fig. 9c), suggesting that immunogenic cell death may be a trigger of the cDC1-initiated adaptive immune response. Despite high numbers of DCs, TILs in the inflamed phenotype over-expressed genes encoding for various immune checkpoints and only a minority of TILs expressed ICOS or 41BB (Fig. 5f). In fact, a large fraction of the inflamed phenotype showed genetic alterations in MHC-I (Fig. 3b) and downregulated expression of MHC-II by tumor cells (Fig. 5d). All the above changes are again inter-related (Supplementary Fig. 9b) and considered part of an immune response-mediated negative-feedback loop, and may contribute to the relatively low frequency of sustained clinical responses to ICI even in the inflamed phenotype.

Our study has a number of limitations. For instance, the predictive value of our classifier is based on relatively small numbers of patients in a phase II trial. In addition, the performance of the gene classifier in other patient subsets, tumor sites, or tumor types, such as those that rely exclusively on TCGA data, might be less accurate and requires validation with IHC. Another limitation is that multiplex-IF analysis was performed using digital image analysis of defined regions which may not fully reflect tumor heterogeneity, and despite vigorous manual verification, computed assignments of compartments and immune cells may harbor a certain degree of misclassification. Last, the proposed spatial phenotype-specific treatments require functional and clinical validation.

In conclusion, our study has resulted in the development and validation of a gene-classifier that accurately assigns spatial immunophenotypes in TNBC and metastatic TNBC, and is associated with prognosis in TNBC and various other cancers. This spatial-phenotype-classifier predicts patient response to anti-PD1 independently of currently used clinical markers and outperformes other gene-signatures, thereby addressing an urgent clinical need. Finally, in-depth analysis of NGS, immunologic and clinical sets of patient data points toward actionable targets that may proof beneficial for phenotype-stratified ICI therapy in TNBC.

## Methods

### Cohorts of patients

Cohort A: Node negative, primary TNBC from patients who did not receive adjuvant treatment. FFPE resection materials were used for: whole tissue stainings for CD8 stainings (*n* = 228); stainings for multiple immune cells/molecules on consecutive sections (*n* = 30); multiplexed stainings for immune effector cells (*n* = 64) and cells/molecules related to spatial phenotypes (*n* = 68); microarray gene expression analysis (*n* = 101, A1); as well as RNAseq data analysis (*n* = 43, A2); Complete clinicopathological records were available with >10-year follow up (*n* = 122).

Cohort B: Node-negative, primary BC from patients who did not receive adjuvant treatment (*n* = 867 of which *n* = 196 basal-like BC) with microarray data retrieved from gene expression omnibus GSE2034, GSE5327, GSE11121, GSE2990, and GSE7390. Details of combined cohort have been described previously^10^.

Cohort C: Primary BC with RNAseq and WGS data (*n* = 347 of which *n* = 66 TNBC)^56^ accessible through European genome-phenome archive EGAS00001001178.

Cohort D: Metastatic TNBC from patients treated with anti-PD1 antibody in the TONIC-trial (*n* = 53 of which *n* = 44 paired samples)^8^ with processed transcriptome data of pre- and postinduction treatment biopsies retrieved via controlled access (available through EGAS00001003535). Stromal TILs were scored independently by RS and HH, according to an accepted international standard from the International Immuno-Oncology Biomarker Working Group (see www.tilsinbreastcancer.org for all guidelines on TIL assessment in solid tumors). PD-L1 stainings (22C3 assay) were assessed independently by RS and HH and the percentage of positive tumor-infiltrating immune cells was scored.

Cohort E: TCGA data^57^, as well as sample annotation data of TNBC were retrieved from the USCS xena browser (*n* = 5194 of which 1284 BC of which in turn 137 TNBC). Transcriptome data of anti-PD1 pretreatment biopsies from melanoma patients (*n* = 28) or treated with anti-PD1 antibody (*n* = 65) were retrieved from GSE78220^36^and GSE91061^35^.

See Supplementary Table 1 and Supplementary Fig. 1 for clinical details and application of these cohorts.

Cohort F: Metastatic TNBC with whole tissue CD8 stainings and RNAseq (*n* = 12 lymph-node macrometastases).

### Ethics statement

This study has been approved by the Medical Ethical Committee at Erasmus MC (MEC.02.953, MEC-2020-0090), and was performed according to the Declaration of Helsinki and the Code for Proper Secondary Use of Human Tissue in The Netherlands (version 2002, update 2011) of the Federation of Medical Scientific Societies in The Netherlands (http://www.federa.org/), the latter granting authorized use of coded spare tissue (from Cohort A and Cohort F) for research. For details on previously published, publicly available datasets see respective references provided in methods and supplementary table 1.

### Tissue stainings and image analysis

#### Immunohistochemistry (IHC)

IHC was performed on TNBC whole tissue sections (FFPE) comprising different histological subtypes, which were assigned by experienced pathologists (Supplementary Fig. 8d). IHC stainings were performed following heat-induced antigen retrieval for 20 min at 95 °C. After cooling to RT, staining was visualized by the anti-mouse EnVision+ ® System-HRP (DAB) (DakoCytomation). The following primary antibodies were used: CD8 (C8/C144B, Sanio, 1:100, pH 9); CD3 (PS1, Sigma, 1:25, pH 6); CD4 (4B12, DAKO, 1:80, pH 9), CD137 (BBK-2, Santa Cruz, 1:80, pH 6), CD278 (SP98, Thermo Fisher, 1:50, pH 9), CD66b (80H3, BIO-RAD, 1:100, pH 9), MECA-79 (C111-6, Santa Cruz, 1:50, pH 9), and MHC-II (LN3, Thermo Fisher, 1:50, pH 9).

#### Multiplexed immunofluorescence (IF)

Multiplexed IF was performed using OPAL reagents (Akoya Biosciences) on whole slides (using a randomly selected subset of cohort A with comparable fractions of all spatial phenotypes). In brief, stainings included multiple cycles of antigen retrieval (15 min boiling in antigen retrieval buffer, pH 6 or pH 9 depending on primary antibodies) followed by cooling, blocking, and consecutive staining with primary antibodies, HRP-polymer, and Opal fluorophores; cycles were repeated until all markers were stained. Finally, nuclei were stained with DAPI.

#### Immune effector panel (number indicates position of primary antibody)

1. CD56 (MRQ-42, Sanbio, 1:500)—OPAL620; 2. CD3 (SP7, Sigma, 1:350)—OPAL520; 3. CD20 (L26, Sanbio, 1:1000)—OPAL650; 4. CD8 (C8/144b, Sanbio, 1:250)—OPAL570; 5. CD68 (KP-1, Sanbio, 1:250)—OPAL540; 6. Cytokeratin-Pan (AE1/AE3, Thermofisher, 1:200)—OPAL690; 7. DAPI.

#### Spatial phenotype panel (number indicates position of primary antibody)

1. CLEC9A (sheep polyclonal*, R&D Systems, 1:600)—OPAL570; 2. S100A7 (47C1068, Biotechne, 1:1000)—OPAL650; 3. CD11b (EP1345Y, Abcam, 1:200)—OPAL690; 4. CD8 (C8/144b, Sanbio, 1:250)—OPAL540; 5. CD163 (MRQ26, Cell Marque, 1:50)—OPAL520, 6. COL10A1 (X53, Life Technologies, 1:50)—OPAL620; 7. Cytokeratin-Pan (AE1/AE3, Thermofisher, 1:200)—Coumarin; 8. DAPI.

* Sheep IgG VisUCyte HRP polymer (R&D Systems) was used as the secondary antibody.

#### Manual scoring

IHC was scored for the frequency of CD8+ T cells at the border and in the center by DH and AMT, independently of each other (illustrated in Supplementary Fig. 2a). The border region included the invasive margin, and covered ~50% tumoral area (tumor cells and stroma) and ~50% peritumoral area (no or only isolated tumor cells, particularly in case of ILC subtypes), whereas the center region included non-necrotic regions, and covered tumor and stroma. In case of LN metastases, only border regions that were not surrounded by lymphoid tissue were evaluated. The spatial phenotype of CD8+ T cells was determined using whole slide scans (Hamamatsu slide scanner) at 1x magnification and using at least 8 regions of interest at 20x magnification in border and center. Scoring criteria were as follows: inflamed: almost equal frequencies of CD8+ T cells at the border and center; excluded: >10 times more CD8+ T cells at the border compared to center; and ignored: hardly any CD8+ T cells present at the border and center. All immune markers stained on consecutive slides were scored at 20x magnification (at border and center) and reported as the percentage of positive cells (of total nuclei). TLS were identified as dense clusters of CD4+ T cells and CD20+ B cells on consecutive slides (as shown in Fig. 1f), whereas HEV were identified as vessels that were MECA-79 positive (frequently found in TLS), and both the TLS and HEV were reported as total number per tumor.

#### Digital image analysis

Following whole slide scans using VECTRA 3.0 (Akoya Biosciences), at least eight stamps (regions of interest; stamp size: 670 × 502 μm^2^; resolution: 2 pixels/μm^2^; pixel size: 0.5 × 0.5 μm^2^) were set in non-necrotic areas at the tumor border (containing 50% peritumoral region) and center (both the comprising tumor, as well as stroma compartments, illustrated in Supplementary Fig. 3b). In case parts of the tissue were disrupted or lost due to repeated staining cycles, fewer stamps were set or tissues were excluded from analysis (in case of <3 stamps at either border or center regions). Tissue-segmentation was performed according to cytokeratin and DAPI staining; cell-segmentation and phenotyping of individual cells were performed according to individual markers and presence of DAPI using Inform software; and enumerations at border (tumor and stroma) and center regions (tumor and stroma) were summarized for all stamps per sample. Spatial phenotypes were determined according to median CD8+ T cell density at border and center as follows: inflamed, >200 cells/mm^2^ at border and ratio between border and center <10; excluded, >200 cells/mm^2^ at border and ratio between the border and center >10; ignored <150 cells/mm^2^ at border and center. All scans fulfilled either of these 3 spatial phenotypes. Collagen-10 was identified through tissue seqmentation and quantified as collagen-10-positive tissue area. Nearest-neighbor analysis was performed in R using the PhenoptR package (Akoya Bioscience), to which end, the number of non-CD8+ T cells within a 10 μm radius of CD8+ T cells were calculated from the Inform-derived cell-segmentation files in Phyton.

### Gene expression and DNA mutational analysis

#### RNA sequencing

RNAseq data were collected from fresh frozen TNBC using 150 bp paired-end with LncRNA library (Ribo-zero RNA) on Illumina HiSeq. RNA was isolated from FFPE using the RecoverAll Total Nucleic Acid Isolation Kit for FFPE (Thermofisher). RNA was sequenced using the FFPE sample Eukaryotic RNA-seq Library (250 ~ 300 bp insert strand specific library with rRNA removal) on the Illumina Novoseq6000 platform at Novogene. Although FFPE starting material yielded poorer quality of RNA when compared to FF samples, we still captured sequencing data of n = 12 out of n = 15 samples with sufficiently high quality: i.e., these samples contained <50% duplicated reads (ranging from 20 to 45%); >50% mapped reads (ranging from 55 to 95%); and expressed >75% of classifier genes.

#### Data normalization

Microarray data were normalized using fRMA^58^ and corrected for batch effects using ComBat^59^. RNAseq data (cohorts A2, C, D E (TNBC)) were aligned with GRCh38 using the STAR algorithm^60^ (version 2.4.2a) and geTMM normalized^61^ for DE analyses. For pan-cancer analyses (Supplementary Fig. 5) preprocessed data was used (i.e., TCGA other than BRCA: EB + + Adjusted; and ICI-treated melanoma patients: FPKM normalized).

#### TCR repertoire, neo-antigen, and mutational signature analysis

TCR clonality was estimated using the MIXCR algorithm^62^; output was processed with tcR package^63^ in R and reported as TCR diversity (total number of TCR-Vβ reads per sample) and TCR repertoire skewness (Gini-Simpson index of TCR-Vβ reads per sample). Prediction of neo-antigens was performed with netMHCv3.4^64^ as described previously^10,65^. In brief, 17-mer peptides containing a mutated amino acid derived from a nonsynonymous mutation at the center position were run through the online prediction server Net-MHC to predict EC50 values of all possible 9-mer peptides for HLA-class I molecules, and a peptide with a predicted EC50 < 50 nM was considered a possible neo-epitope. Mutational signatures were identified through the Wellcome Trust Sanger Institute mutational signatures framework^56,66^.

#### Differential gene-, pathway-, and immune cell subset analyses

Differential gene expression (DE) analysis was performed in R using limma/voom. Differentially expressed genes (*p* < 0.05, logFC > 1) were used for ingenuity pathway analysis (IPA software, core analysis). Spatial phenotypes were also interrogated for DE of gene-sets related to T cell evasion^10^. The expression of a gene-set was determined as an average expression of all genes in the respective set. Immune cell frequencies were estimated using the CIBERSORT algorithm^67^ in absolute mode. Gene-set entichment analysis for *Hallmark* and *Kegg* datasets (v7.2) was performed using GSEA 4.1.0 software^68^ using weighted signal-t- noise ranking with 1000 permutations.

### Gene classifier to assign spatial phenotypes

In a discovery set (Cohort A1, *n* = 101 primary TNBC), we selected the top differentially expressed genes among inflamed, excluded and ignored phenotypes (>1logFC among all 3 phenotypes; *p*_adj_ < 0.05) of samples with microarray data and corresponding CD8+ T cell staining data (Fig. 2a). Expressions for each classifier gene were averaged for each of the three spatial phenotypes, ranks of gene expressions were calculated per spatial phenotype (Supplementary Data 1), and assignments were based on the highest Spearman rank-correlations between the unknown samples and ranked expressions of classifier genes per spatial phenotypes of the discovery set. In a validation set (Cohort A2, *n* = 43 primary TNBC), RNAseq data of independent samples with corresponding CD8+ T cell staining data were used to assign phenotypes based on the highest rank-correlations with the discovery set (A1), and yielded 81% accuracy (Table 1). Correct assignment of unknown samples from Cohort B (RNAseq data) was verified by comparison of T cell characteristics, such as TCR-Vβ repertoire diversity and numbers of intratumoral T cells, with those of Cohort A2 (RNAseq and CD8+ T cell stainings), and the classifier-assigned samples were found non-different compared to those from the validation set (Supplementary Fig. 4a, b). An additional validation set (Cohort F, *n* = 12 metastasized TNBC) with RNAseq data and corresponding CD8+ T cell staining data showed 83% accurate assignment of spatial immunophenotypes in TN lymph-node metastases (Table 2). Clinical validation was done using metastasized lesions from TNBC patients treated with anti-PD1 antibody (Cohort D, pretreatment), from which 3 out of 53 samples were excluded because of equally high rank correlations. Assignment of spatial phenotypes in metastatic lesions did not depend on lesion site. We did observe significantly different proportions (decreased frequency of inflamed, as expected) in the metastasized (cohort D) versus primary setting (Cohorts A1 and C) (Supplementary Fig. 4d, e). Predictive value of classifier gene-sets was determined by fitting ROC curves for anti-PD1 response. Responders (CR, PR, SD > 24 weeks) and nonresponders (PD) were separated using the pROC package in R. Excluded and inflamed gene-sets were calculated as average scores of all respective genes, and PD-L1 and sTIL scores were scored as described before^8^.

### Statistical analysis

Statistical analysis was performed in R version 3.5.1 or GraphPad Prism 6. Log-rank test for trend was used to compare Kaplan–Meier curves; Cox-regression analysis was used to assess HR of immunophenotypes, clinical parameters (age, grade, and size which were used as continuous variables), cell types or gene-sets; and Logistic regression was used to determine OR of gene-sets (glm.OR function). Multiple testing correction was performed for differential gene expression analysis using the Benjamini–Hochberg method. Kruskal–Wallis test was used to assess differences in gene expression and immune cell densities among spatial phenotypes; Pearson-correlation was used to assess linear relationships between continuous variables; and Chi-Square test or Fishers’ exact test (in case of small sample sizes) were used to assess relationships among factorial variables. The following significance levels were used: *p < 0.05; **p < 0.01; ***p < 0.001; ****p < 0.0001; NS, *p* > 0.5.

### Reporting summary

Further information on research design is available in the Nature Research Reporting Summary linked to this article.

## Supplementary information

Supplementary Information

Reporting Summary

Supplementary Dataset 1

Supplementary Dataset 2

Supplementary Dataset 3

Peer Review File

Description of Additional Supplementary Files

## Data Availability

All WGS, RNAseq and microarray data are available at the Gene-Expression Omnibus (GEO), European-Genome Phenome Archive and USCS Xena browser: RNAseq data from Cohort A is available through at GEO (GSE177043). Microarray data from Cohort A is available at GEO (GSE12276; GSE47389 and GSE27830, for individual sample accession codes see Supplementary Data 2). For processed imaging and immunogenomics data of Cohort A see Supplementary Data 2. Raw imaging data is available upon request. Microarray data from Cohort B is available at GEO (GSE2034, GSE5327, GSE2990, GSE11121 and GSE7390^69–73^). RNAseq data from Cohort C is available at EGA (EGAS00001001178^56^). RNAseq data from Cohort D is available under controlled access at EGA (EGAS00001003535^8^). Cohort E has been retrieved from https://xenabrowser.net/ (data generated by the TCGA Research Network: https://www.cancer.gov/tcga.), and from GEO (GSE78220, GSE91061^35,36^). Classifier gene expressions and IHC scores from Cohort F are provided in Supplementary Data 3. The remaining data are available within the article, supplementary information or source data file. Source data are provided with this paper.

## Source data

Source Data
