# Supplementary material for: Spatial immunophenotypes predict response to anti-PD1 treatment and capture distinct paths of T cell evasion in triple negative breast cancer
Source: Nat Commun. 2021 Sep 27;12:5668. doi: 10.1038/s41467-021-25962-0 (PMC8476574; doi:10.1038/s41467-021-25962-0)
Supplement: Supplementary file 1 — Supplementary Information [file 41467_2021_25962_MOESM1_ESM.pdf]

Supplementary Tables and Figures

Cohorts	Cohort A	Cohort B	Cohort C	Cohort D	Cohort E	Cohort F
**Tumor types and number of patients**	n=228 TNBC	n=867 BC of which n=196 basal-like	n=347 BC of which n=66 TNBC	n=53 TNBC from phase II clinical trial (n=44 paired samples from metastatic lesions pre- and post-induction treatment)	n=1284 BRCA of which n=137 TNBC; n=477 SKCM; n=454 BLCA; n=877 LUAD; n=623 PRAD; n=567 HNSC; n=91 KICH; n=309 CESC; n=183 PAAD; n=329 COAD; n=81 ICI treated metastatic SKCM	n=12 TN lymph node macro-metastases
**Disease stage***	primary	primary	primary	metastatic	primary	metastatic
**Treatment***	none	none	none	anti-PD1 preceded by 2-weeks cisplatin (n=9); cyclophosphamide (n=12); doxorubicin (n=11); irradiation (n=10); or no induction (n=11)	variable	variable
**Age (mean)***	52	53	54	51	58	NA
**Tumor size***	33% T1; 47% T2; 4% T3; 1% T4; 15% NA	39% T1; 60% T2; 0.5% T3; 0.5% T4	24% T1; 37% T2; 5%T3; 4% T4; 30% NA	NA	NA	NA
**Nodal status***	70% LN0; 16% LN+; 14% NA	100% LN0	45% LN0; 24% LN+; 31% NA	all M1	65% LN0; 35% LN+	100% LN+
**Datasets**	prognosis discovery: n=122 (LNN with clinical records >10 year follow up); gene classifer discovery: n=101 (microarray); classifier validation: n=43 primary, immune effector panel: n=64; spatial phenotype panel: n=69; IHC (multiple markers): n=30	prognosis validation: n=786 (microarray and clinical records)	genomic features: n=347 (WGS and RNAseq)	predicton: n=53 (RNAseq, PD-L1 staining, clinical records)	prognosis/prediction (RNAseq, clinical records, anti-PD1 response)	Validation of gene-classifier: RNAseq from FFPE
**Determination of spatial immunophenotypes**	CD8 stainings	gene-classifier	gene-classifier	gene-classifier	gene-classifier	CD8 stainings
**Access**	GSE177043, Supplementary file 2	GSE2034, GSE5327, GSE11121, GSE2990, GSE7390	EGAS00001001178	EGAS00001003535	https://xenabrowser.net/, GSE7822036, GSE9106135	Supplementary file 3

Supplementary Table 1. Overview of study cohorts. Clinical features and access of the different cohorts. ***characteristics for TNBC patients. Abbreviations: LN0: lymph-node negative; LN+: lymph-node positive; NA: not available; BRCA: breast cancer; BLCA: bladder cancer; SKCM: skin-cutaneous melanoma; LUAD: lung adenocarcinoma; HNSC: head and neck squamous-cell carcinoma; PRAD: prostate adenocarcinoma; PAAD: pancreatic adenocarcinoma; COAD: colorectal adenocarcinoma; CESC: cervical squamous cell carcinoma and endocervical adenocarcinoma; KICH: chromophobe renal cell carcinoma.

a	Covariate	HR (CI)	p-value
	inflamed immunophenotype based on IHC	0.31 (0.13-0.75)	0.009
	tumor size	1.01 (0.98-1.05)	0.19
	grade	0.89 (0.49-1.6)	0.69
	age	0.98 (0.95-1.01)	0.13
	TLS presence	0.64 (0.29-1.5)	0.28
	TLS frequency	1.00 (0.78-1.17)	0.13

b	Covariate	HR (CI)	p-value
	inflamed immunophenotype based on gene expression	0.17 (0.05-0.58)	0.004
	nodal status	6.83 (0.85-54.8)	0.07
	tumor size	1.17 (0.4-3.39)	0.76
	age	1.02 (0.98-1.05)	0.23
	TLS signature (Carbrita, Nature, 2020)	1.3 (0.7-2.5)	0.38

Supplementary Table 2. Multivariable Cox regression analysis including spatial immunophenotypes. a. Multivariable Cox regression analysis including spatial immunophenotypes based on IHC (Cohort A, n=106). **b**. Multivariable Cox regression analysis including spatial immunophenotypes based on gene-expression (Cohort E, n=140). P-values correspond to cox-proportional hazard model. All spatial immunophenotypes were part of analysis, but outcomes are displayed for inflamed immunophenotype. Abbreviations: TLS: tertiairy lymphoid structures, HR: hazard ratio, CI: confidence interval.

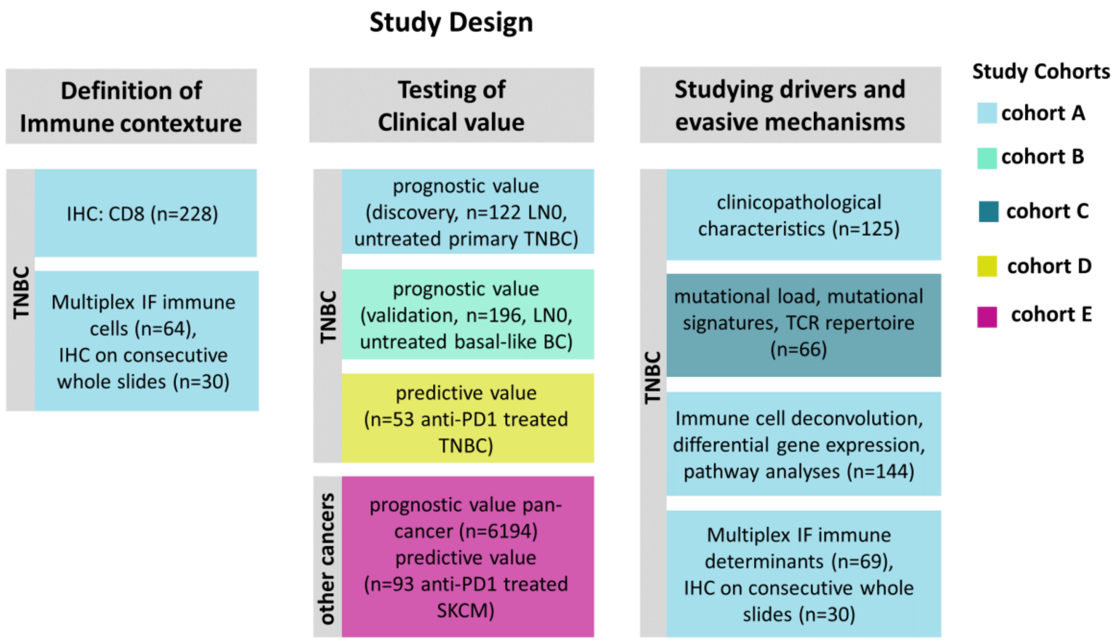

Supplementary Fig. 1 Study Design. Different steps and types of analyses regarding spatial immunophenotypes. Colors of boxes reflect the cohorts used for each step (for details and clinical characteristics of cohorts see M&M section and **Supplementary Table 1**). For cohort A spatial phenotypes were identified using IHC of CD8+ T-cells on whole slides and for cohort B-E spatial phenotypes were assigned using the gene-classifier

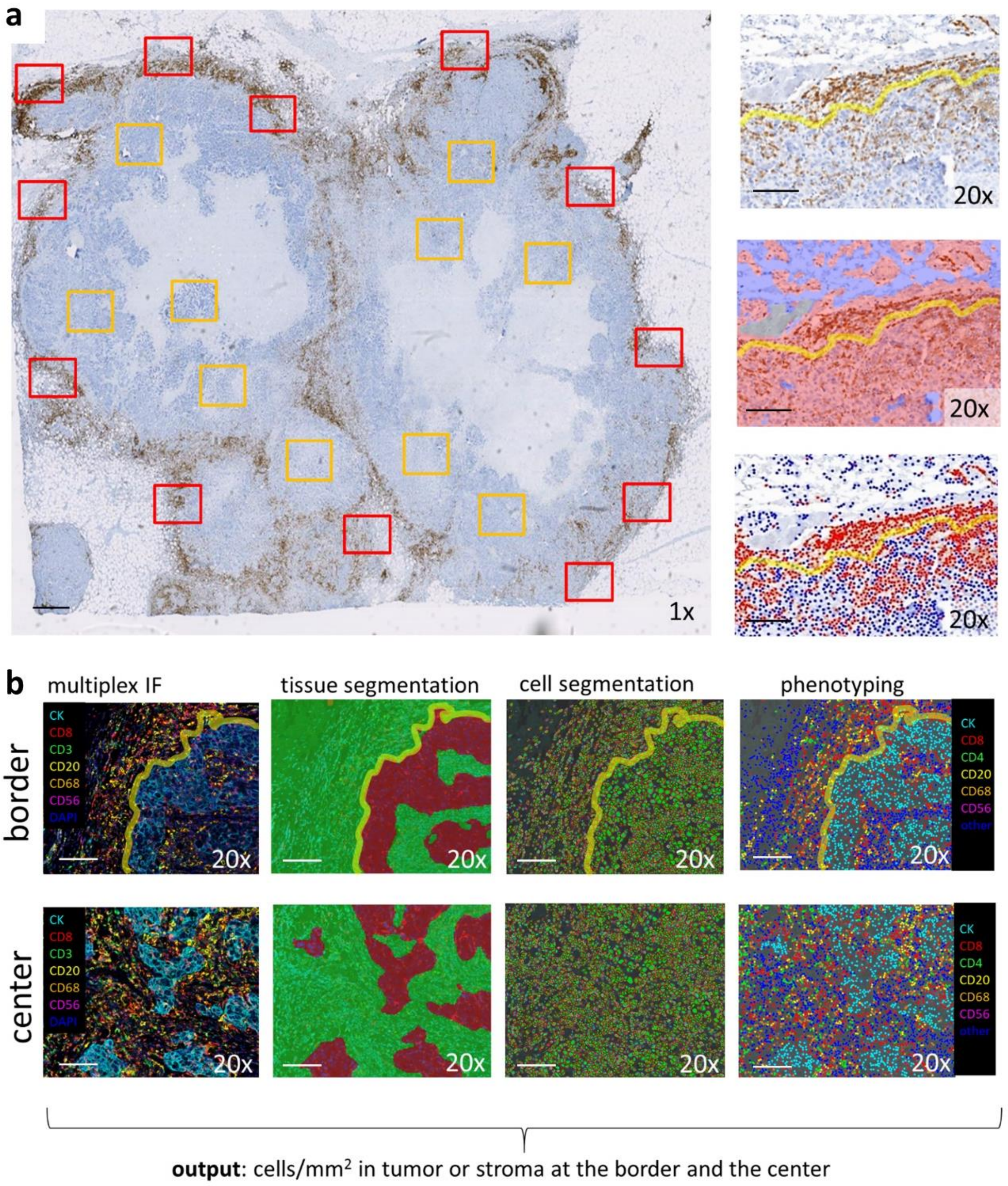

Supplementary Fig. 2. Workflow for digital image analysis of immune stainings. a. Whole slide images of CD8+ T-cell IHC with border and center stamps (regions of interest, red and yellow, respectively) with close-up (20x magnification, scalebar represents 100μm) of one border stamp (top), separation of tissue (red) and empty space (blue) (middle) and identification of CD8-positive (red) and negative (blue) cells (bottom). Yellow line indicates outer tumor margin. **b.** Image analysis for multiplex IF of immune effector cell panel at border and center of an inflamed TNBC; from left to right: multicolour IF image, tissue segmentation (red: tumor; green: stroma; orange: empty space, yellow line: outer tumor margin); cell segmentation; and individually phenotyped cells (Inform software). Scalebar represents 100μm. Stamp size: 670x502 μm^2; resolution: 2 pixels/μm^2; pixel size: 0.5x0.5 μm^2.

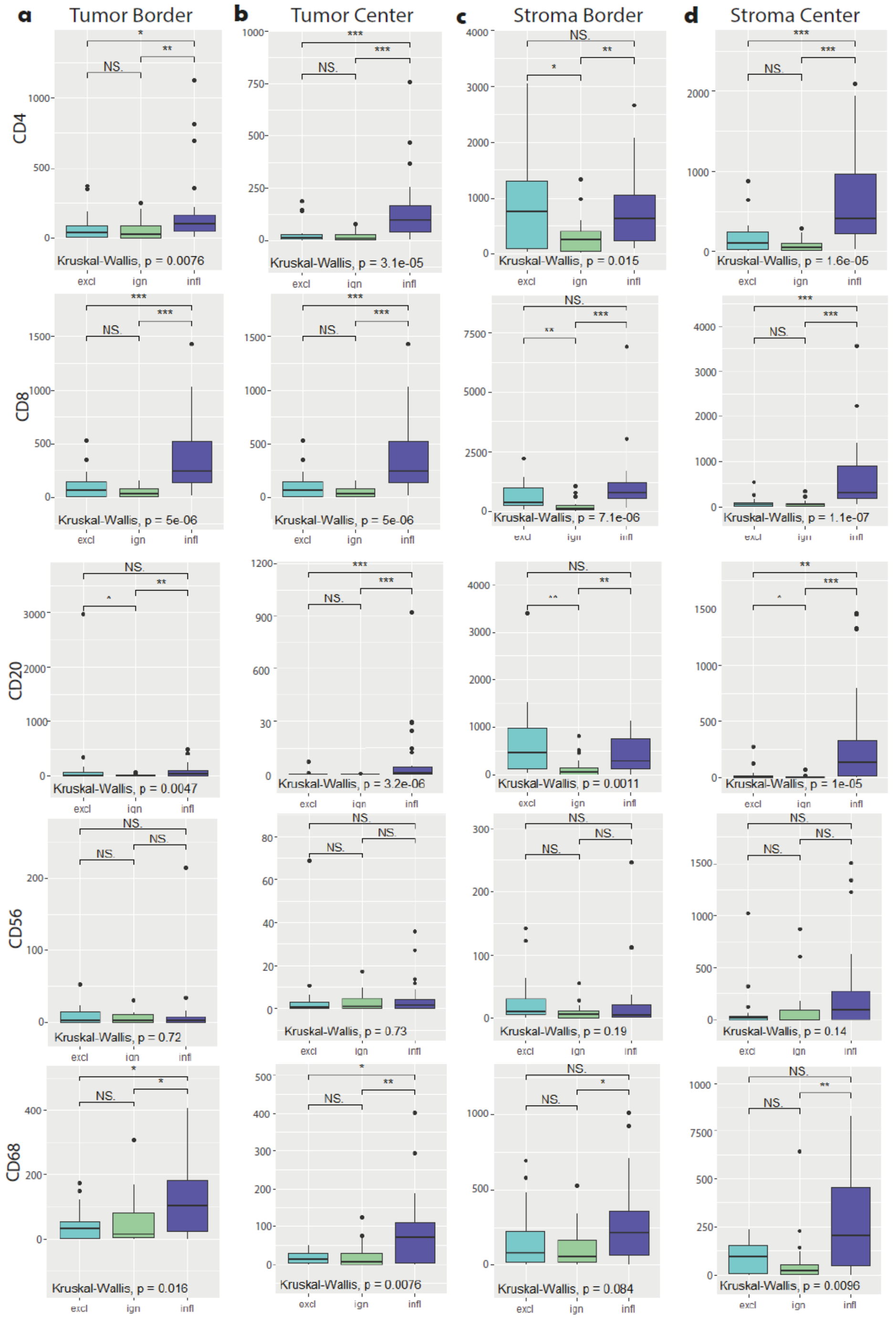

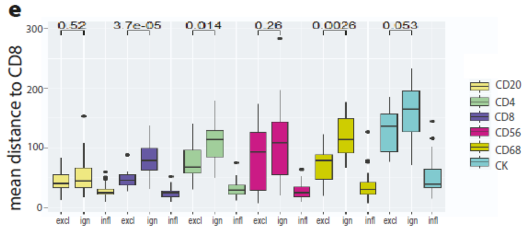

f

	Border stroma	Border tumor	Center stroma	Center tumor
**CD8**	**0.34 (0.12-0.95)**	**0.33 (0.12-0.94)**	0.45 (0.17- 1.2)	0.43 (0.16-1.1)
**CD4**	0.36 (0.13-1)	**0.24 (0.07-0.72)**	0.76 (0.3-1.9)	0.78 (0.31-2)
**CD20**	0.52 (0.2-1.4)	0.43 (0.16-1.2	0.58 (0.36-2.3)	**0.36 (0.13-0.95)**
**CD68**	0.6 (0.23-1.5)	0.68 (0.26-1.7)	0.9 (0.36-2.3)	0.82 (0.32-2.1)
**CD56**	1.6 (0.62-4.1	2 (0.74-5.3)	1.9 (0.72-5.1)	2 (0.75-5.3)

Supplementary Fig. 3. Immune effector cells according to spatial phenotypes in TNBC. Boxplots show median with 25th-75th percentile and range (outliers are displayed as dots) for cell densities (in cell number/mm^2) following staining and analysis using immune effector panel. **a.** Tumor border. **b.** Tumor center. **c**. Stroma border. **d.** Stroma Center. **e**. Boxplots show median with 25th-75th percentile and range (outliers are displayed as dots) for distances between CD8+ T-cells and other cell types. **f**. Table with Hazard Ratios (HR) and 95% confidence intervals (CI, between brackets) for MFS of immune cell densities (significant HR values are shown in bold, analyses not corrected for multiple testing). Cohort A was used for panels **a-f**, n=64 LNN TNBC of which n=18 with

exluded-, n=19 with ignored- and n=27 with inflamed phenotype). Significant differences are: ***, p<0.001; **, p<0.01; *, p<0.05; NS, p>0.5 (Kruskal-Wallis, one-sided). Source data are provided within source data file.

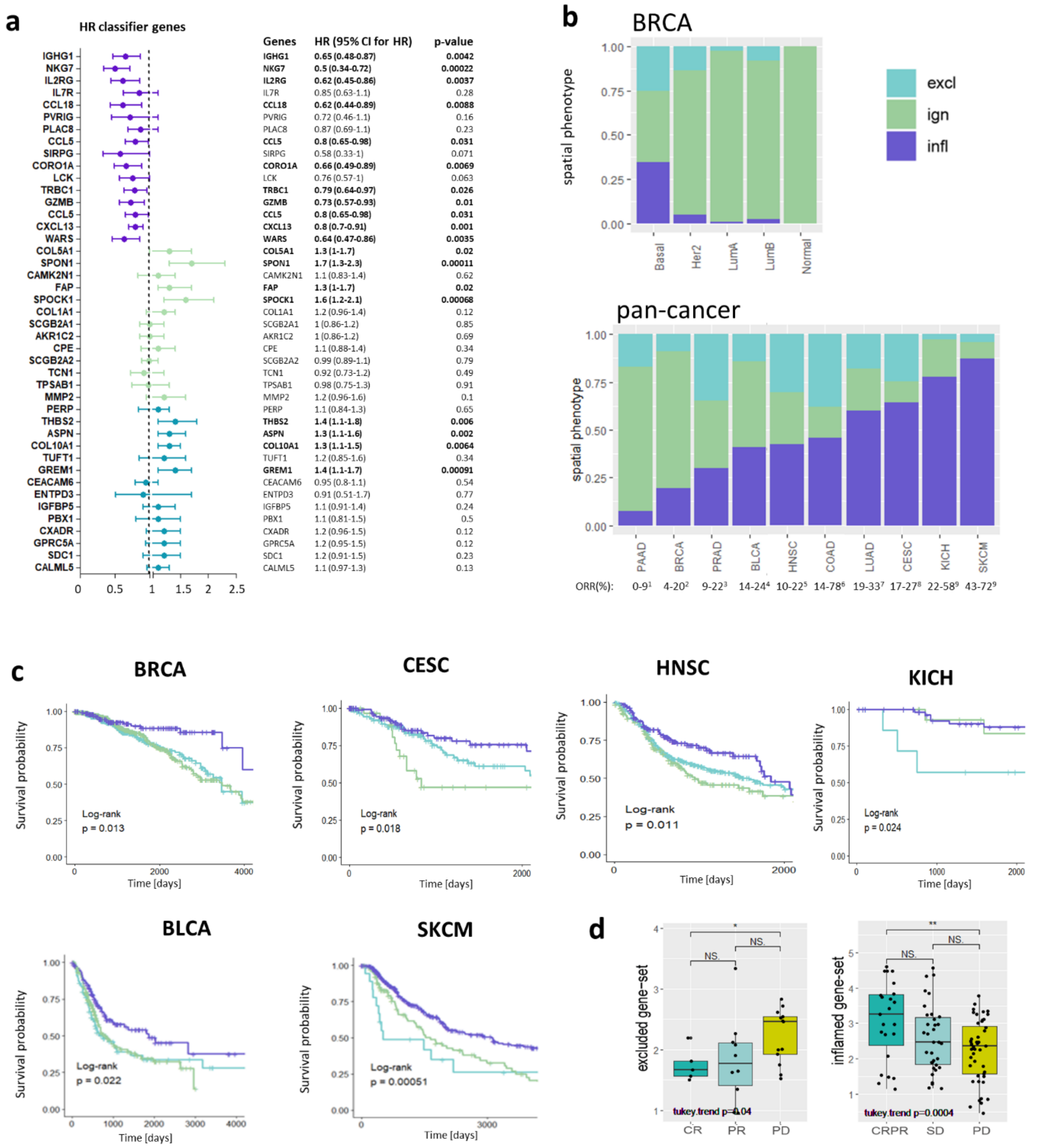

Supplementary Fig. 4. Spatial phenotypes in non-TNBC cancers. a. Forestplots showing HRs and CIs (error bars) of individual classifier genes (Cohort B, not corrected for multiple testing, n=196 basal-like BC). **b**. Stacked bar-graphs show frequencies of spatial phenotypes in different breast cancer subtypes (left, Cohort B, n= 867 BC) and various cancer types (right, Cohort E, n=5194, see Table S1 for details); below right panel objective response rates (ORR) are listed for ICI treatments of respective cancer types [1,2,3,4,5,6,7,8,9] (see References). **c.** Kaplan-Meier curves for OS stratified per spatial phenotype in various cancer types (Cohort E, p-value shows two-sided log-rank test). **d**. Boxplots displaying median with 25th-75th percentile and range (outliers are displayed as dots) for average expression of gene-sets for the excluded as well as inflamed phenotype in responding and non-responding melanoma patients following ICI treatment (left, Hugo Cohort, n=28 SKCM of which n=5 CR, n=10 PR and n=13 PD; right, Riaz Cohort, n=105 SKCM of which n=23 PRCR, n=34 SD and n=48 PD). Significant differences are: **, p<0.01; *, p<0.05; NS, p>0.5 (Tukey trend test). Abbreviations: PAAD: pancreatic adenocarcinoma; BRCA: breast carcinoma; PRAD: prostate adenocarcinoma; BLCA: bladder urothelial carcinoma; HNSC: head and neck squamous cell carcinoma; COAD: colon adenocarcinoma; LUAD: lung adenocarcinoma; CESC: cervical squamous cell carcinoma and endocervical adenocarcinoma; KICH: chromophobe renal cell carcinoma; SKCM: skin cutaneous melanoma. Source data are provided within source data file.

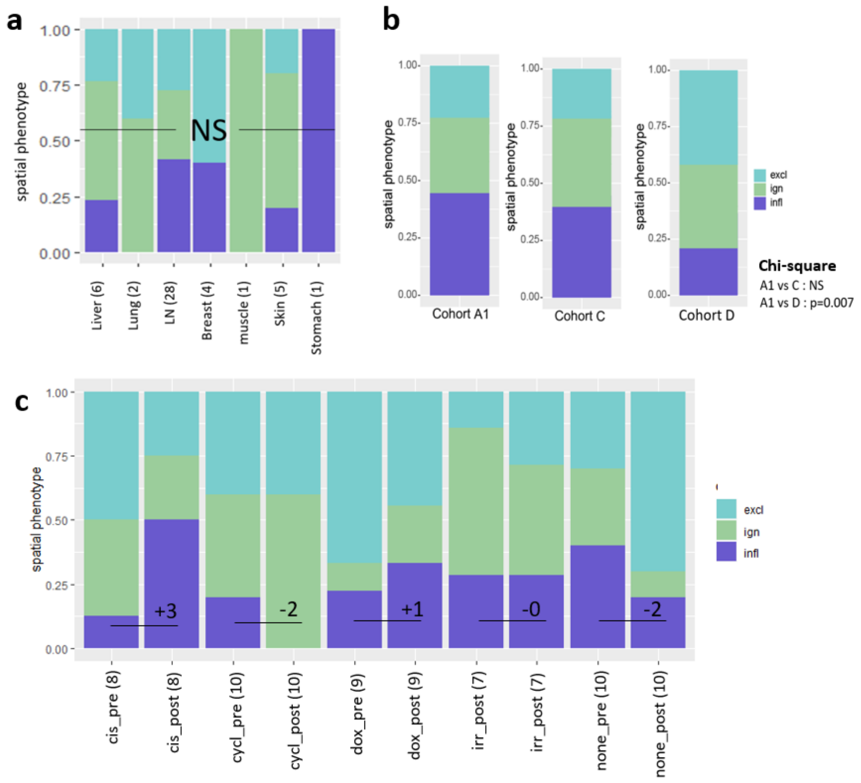

Supplementary Fig. 5. Spatial phenotypes in metastasized TNBC according to distant sites and induction treatment. **a**. Stacked bar graphs show frequencies of spatial phenotypes assigned via gene classifier in different metastatic lesions (number indicates total number of lesions, n=47). **b**. Frequencies of spatial phenotypes in Cohort A1 (CD8 stainings, primary tumors, n=101), Cohort C (gene-classifier, primary tumors, n=66) and Cohort D (gene-classifier, metastatic lesions, n=51). **c**. Paired frequencies of spatial phenotypes pre- and post-induction treatments (number indicates a change to inflamed phenotype, n=44 paired mTNBC). Significant differences are: ***, p<0.001; **, p<0.01; *, p<0.05; NS, p>0.05 (Chi-square test). Abbreviations: cis: cisplatin; cycl: cyclophosphamide; dox: doxorubicin; irr: irradiation; none: no induction.

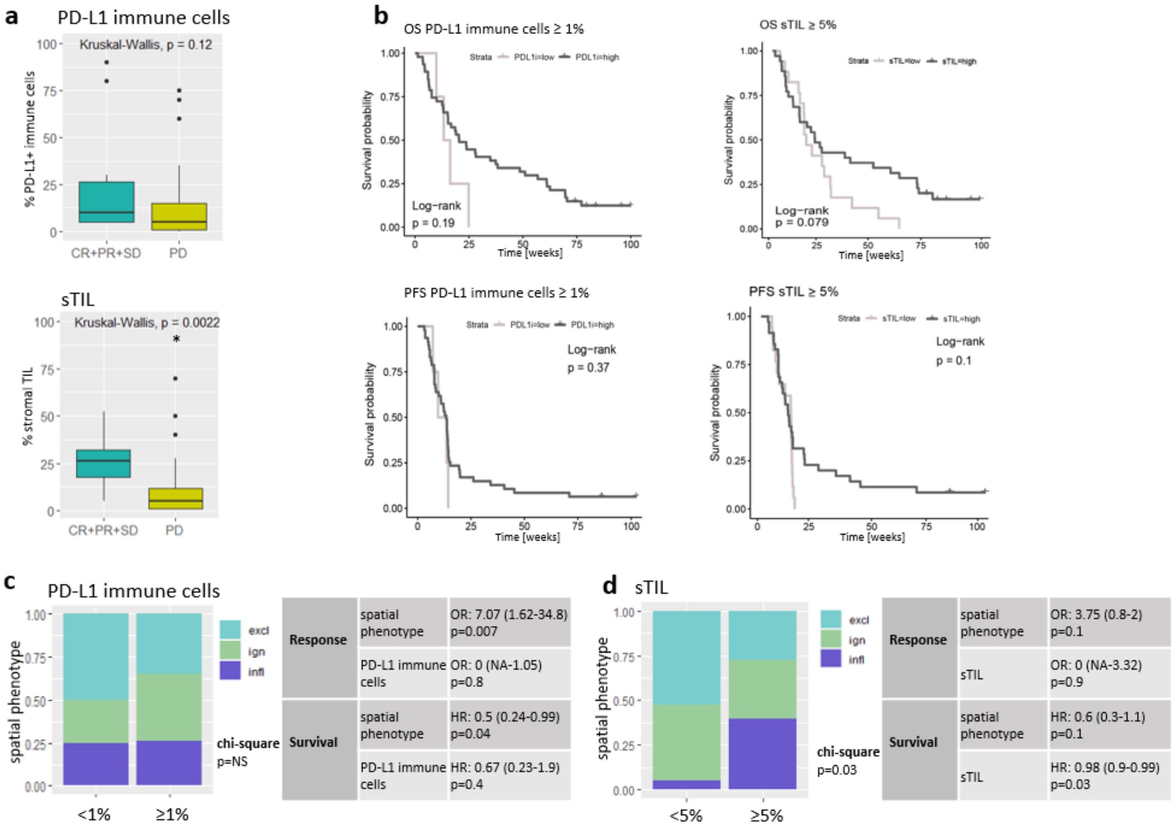

Response	spatial phenotype	OR: 7.07 (1.62-34.8) p=0.007
	PD-L1 immune cells	OR: 0 (NA-1.05) p=0.8
Survival	spatial phenotype	HR: 0.5 (0.24-0.99) p=0.04
	PD-L1 immune cells	HR: 0.67 (0.23-1.9) p=0.4

Response	spatial phenotype	OR: 3.75 (0.8-2) p=0.1
	sTIL	OR: 0 (NA-3.32) p=0.9
Survival	spatial phenotype	HR: 0.6 (0.3-1.1) p=0.1
	sTIL	HR: 0.98 (0.9-0.99) p=0.03

Supplementary Fig. 6. Standardly used predictive markers of ICI response in patients with metastatic TNBC. a. Boxplots show median with 25th-75th percentile and range (outliers are displayed as dots) for fraction of PDL1-positive immune cells (upper plot) and fraction of stromal TIL (sTIL) per response group (all Cohort D, n=50 mTNBC, of which n=10 CR+PR+SD and n=40 PD; p-value shown for Kruskal-Wallis, one-sided). **b**. Kaplan-Meier curves for OS and PFS with different cutoffs (≥1%, left) for PDL1 and sTIL (≥5%) (p-values shown for log-rank test). **c**. Stacked bar graphs show frequencies of spatial phenotypes stratified by immune cell PD-L1≥1% (p-value shown for chi-square test); table shows spatial phenotypes and immune cell PD-L1 in multivariable models according to prognostic value (hazard ratio (HR), 95% confidence interval (CI) between brackets and p-value) as well as predictive value (odds ratio (OR), 95% CI and p-value, n=49 mTNBC of which n=10 CR+PR+SD and n=39 PD). **d**. Stacked bar graphs show frequencies of spatial phenotypes stratified by sTIL≥5% (p-value shown for chi-square test); table shows spatial phenotypes and sTIL in multivariable models according to prognostic value (HR, CI and p-value) as well as predictive value (OR, CI and p value, n=49 mTNBC of which n=10 CR+PR+SD and n=39 PD).

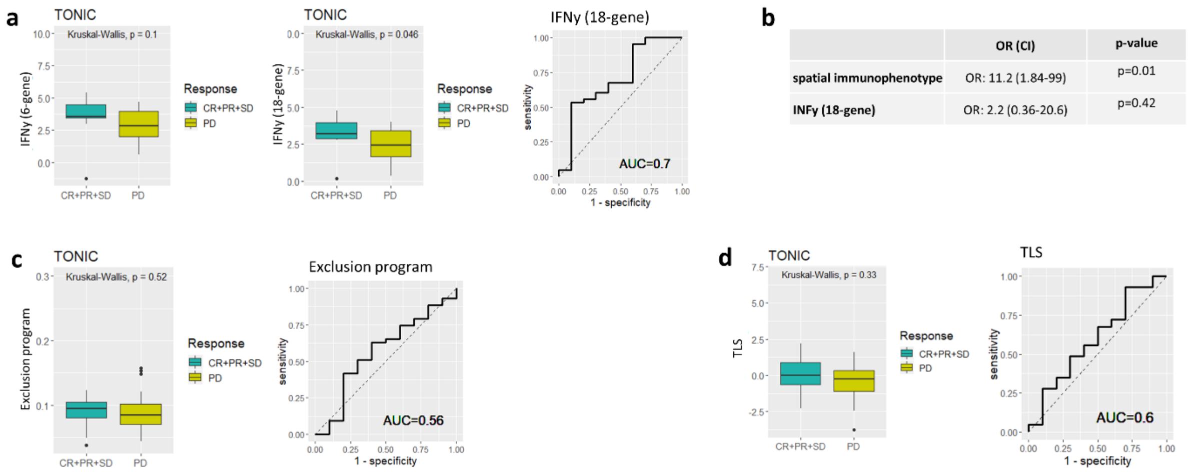

	OR (CI)	p-value
**spatial immunophenotype**	OR: 11.2 (1.84-99)	p=0.01
**INFγ (18-gene)**	OR: 2.2 (0.36-20.6)	p=0.42

Supplementary Fig. 7. Predictive value of spatial immunophenotype gene classifier versus public classifiers. a. Box-plots display median with 25th-75th percentile and range (outliers are displayed as dots) for signature scores in responder (CR+PR+SD) and non-responder patients (PD) from TONIC trial according to a short (6-gene) and extended (18-gene) interferon gamma signature from ref[10]. ROC displays area under the curve for predicting anti-PD1 response (CR+PR+SD) using the extended signature. **b.** Multivariable analysis including spatial immunophenotype gene-classifier and the extended IFNγ signature. **c**. Box plots and ROC according to a T cell exclusion program signature from ref[11]. **d.** Box plots and ROC according to a tertiary lymphoid structure signature from ref[12]. Cohort D was used for panel **a-d** (n=53 mTNBC of which n=10 CR+PR+SD and n=43 PD). Statistical significance was assessed using Kruskal-Wallis, one-sided. Source data are provided within source data file.

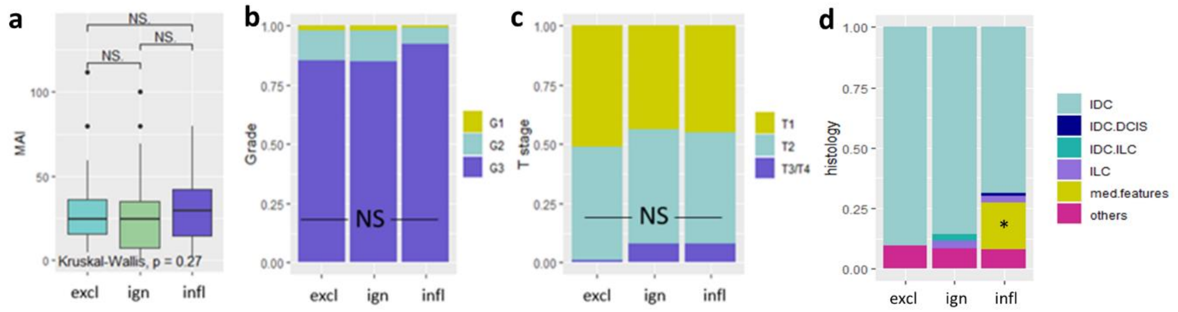

Supplementary Fig. 8. Clinicopathological features of spatial phenotypes. a. Bpxplots display median with 25th-75th percentile and range (outliers are displayed as dots) for mitotic activity index (MAI). **b**. Tumor grade. **c**. Tumor stage. **d**. Histological subtypes. Significant differences are indicated: *, p<0.5, NS, p>0.5 (Chi-square). Cohort A was used for panels **a-d**, n=120 LNN TNBC of which n=37 exluded, n=27 ignored and n=56 inflamed. Source data are provided within source data file. Abbreviations: DCIS: ductal carcinoma in situ; IDC: invasive ductal carcinoma; ILC: invasive lobular carcinoma; med. features: medullary features.

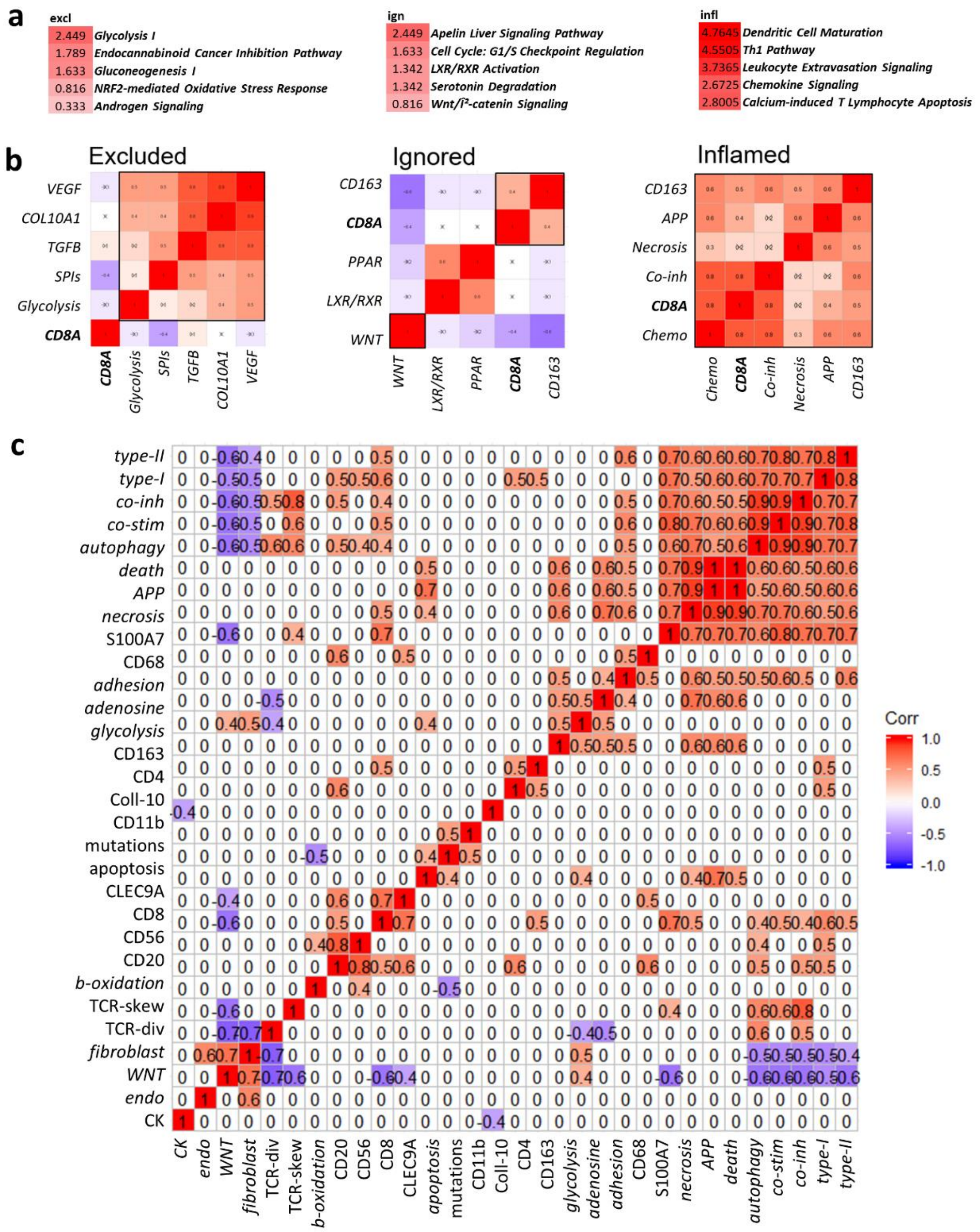

Supplementary Fig. 9. Immune determinants of spatial phenotypes and their inter-relationships in TNBC. a. TOP differentially up-regulated canonical pathways per spatial phenotype (IPA, based on DE analyses, cohort A, n=144 LNN TNBC). **b.** Correlation matrices of pathways in relation to CD8+ T-cells per spatial phenotype (Pearson-correlation; red: positive correlation; blue: negative correlation; crossed R-value: insignificant correlation, Cohort A, n=101). **c.** Integrated correlation matrix of pathway and immune contextures of matched samples (n=30, cohort A; red: significant positive correlation blue: significant negative correlation; white = no correlation). Abbreviations: SPI: serine protease inhibitor gene-set; CK; cytokeratin; endo: endothelial cell barrier gene-set; WNT: WNT-signaling gene-set; fibro: fibroblast barrier gene-set; TCR-div: TCR repertoire diversity; TCR-skew: TCR repertoire clonality (gini-simpson index); b-oxidation: beta-oxidation gene-set; CD20: CD20+ B cells/mm^2; CD8: CD8+ T cells/mm^2; CLEC9A: CLEC9A+ DC/mm^2; apoptosis: apoptosis gene-set; mutations: total number of expressed mutations; CD11b: CD11b+ cells/mm^2, COL10A1: collagen-10 area, CD4: CD4+ T cells/mm^2; Cd163: CD163+ cells/mm^2; glycolysis: glycolysis gene-set; ado: adenosine signalling gene-set; adhesion: cell adhesion signalling gene-set; CD68: CD68+ cells/mm^2, S100A7: S100A7+ cells/mm^2, AP: antigen-processing and presentation gene-set, autophagy: autophagy gene-set; costim: T cell co-stimulation gene-set;

chemo: T cell chemokine gene-set; coinh: T cell co-inhibition gene-set; type-I: type-I interferon gene-set; type-II: type-II interferon gene-set.

a

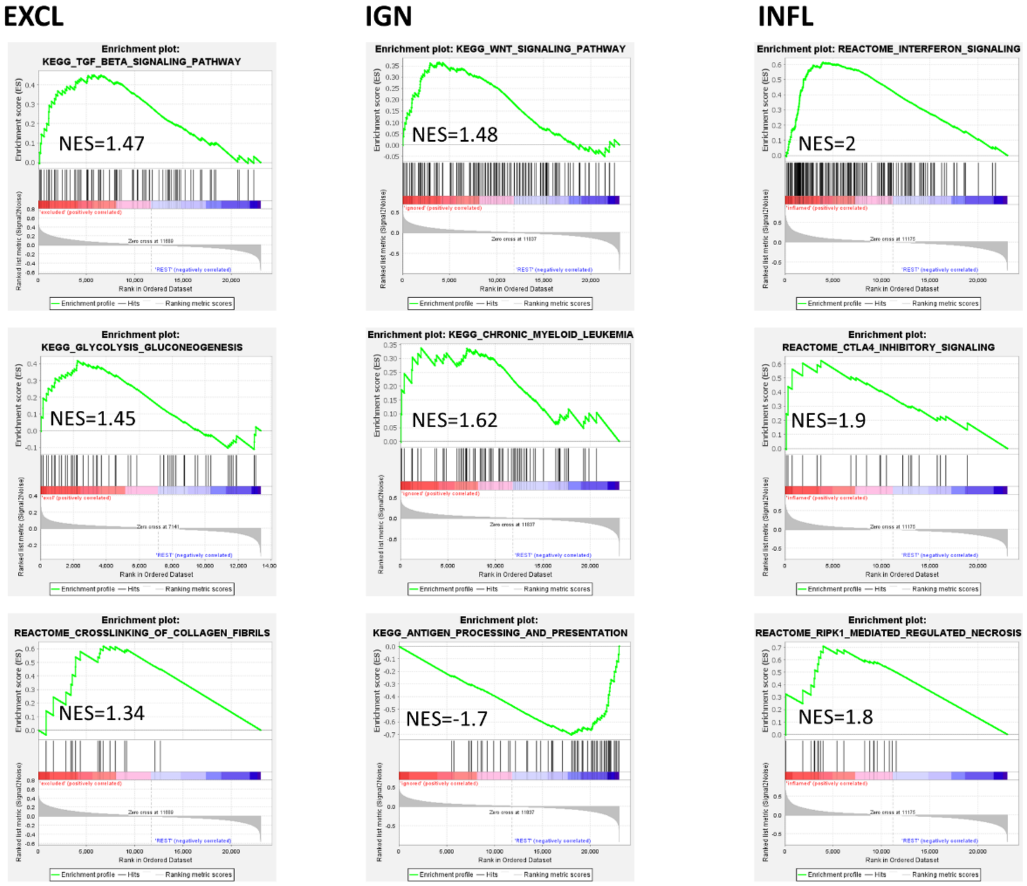

b

Excluded

KEGG	NES	REACTOME	NES
TGF_BETA_SIGNALING_PATHWAY	1.478134	*CRMPS_IN_SEMA3A_SIGNALING*	1.6600893
MELANOMA	1.400128	*FGFR2_MUTANT_RECEPTOR_ACTIVATION*	1.6470528
TASTE_TRANSDUCTION	1.398243	*FRS_MEDIATED_FGFR2_SIGNALING*	1.6412201

Ignored

KEGG	NES	REACTOME	NES
STEROID_BIOSYNTHESIS	1.703354	*MAPK_TARGETS_NUCLEAR_EVENTS*	1.7724794
VASOPRESSIN_REGULATED_WATER_REABSORPTION	1.688975	*MET_PROMOTES_CELL_MOTILITY*	1.7694103
FOCAL_ADHESION	1.633313	*SYNAPTIC_ADHESION_LIKE_MOLECULES*	1.7659823

Inflamed

KEGG	NES	REACTOME	NES
ALLOGRAFT_REJECTION	1.82267	*TNF_SIGNALING*	2.068332
AUTOIMMUNE_THYROID_DISEASE	1.835758	*REGULATION_OF_IFNA_SIGNALING*	2.0640402
GRAFT_VERSUS_HOST_DISEASE	1.838929	*REGULATION_OF_TNFR1_SIGNALING*	2.053227

Supplementary Fig. 10. Gene-set enrichment analysis for spatial immunophenotypes in TNBC. a. Enrichment plots from KEGG and REACTOME databases showing those gene-sets and pathways that are specifically enriched in the excluded (left panel, n=14), ignored (middle panel, n=13) and inflamed (right panel, n=16) phenotypes in LNN TNBC (Cohort A), and which have also been identified using DE and IPA analysis (see **Fig. 4**). **b**. Top 3 enriched pathways according to KEGG and REACTOME databases with normalized enrichment scores (NES) per spatial immunophenotype.

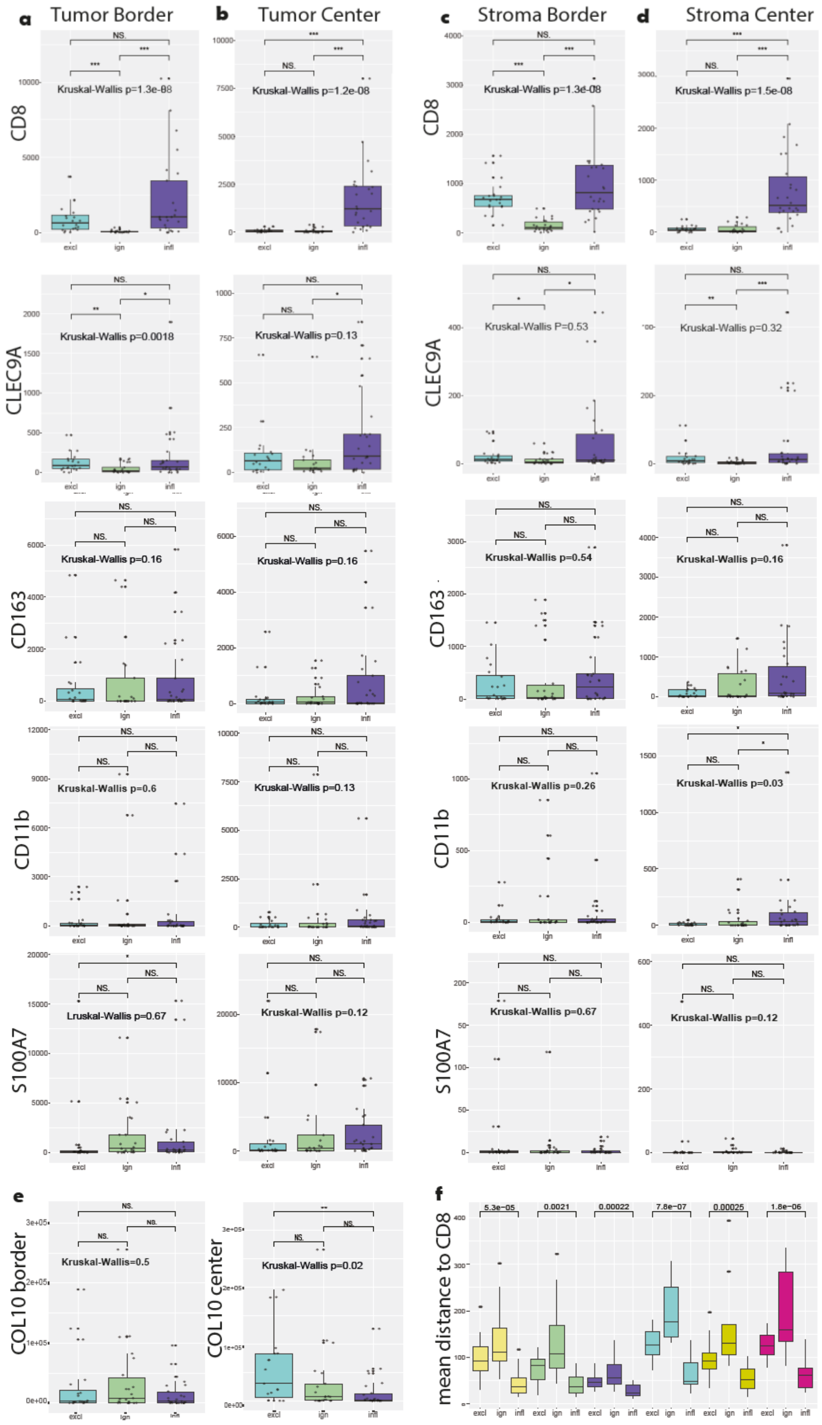

g

	Border stroma	Border tumor	Center stroma	Center tumor
**CD8**	0.42 (0.15-1.2)	**0.34 (0.12-0.98)**	0.46 (0.17-1.2)	0.46 (0.17-1.3)
**CLEC9A**	0.38 (0.13-1.1)	1.3 (0.5-3.5)	0.53 (0.2-1.4)	0.59 (0.23-1.6)
**CD11b**	0.69 (0.26-1.8)	0.48 (0.18-1.3)	0.46 (0.17-1.2)	0.5 (0.19-1.3)
**CD163**	0.51 (0.19-1.4)	**0.35 (0.12-1)**	0.37 (0.13-1.1)	0.71 (0.27-1.9)
**S100A7**	1.4 (0.52-3.6)	**0.26 (0.085-0.8)**	0.87 (0.33-2.3)	0.48 (0.18-1.3)

Supplementary Fig. 11. Immune determinants according to spatial phenotypes in TNBC. Boxplots show median with 25th-75th percentile and range (outliers are displayed as dots) for cell densities (cell number/mm^2) following staining and analysis using spatial phenotype panel. **a.** Tumor border. **b.** Tumor center. **c**. Stroma border. **d.** Stroma Center. **e.** boxplots show median with 25th-75th percentile and range (outliers are displayed as dots) for collagen-10 area per phenotype. **f**. Boxplots show mean distances between CD8+ T-cells and other cell types. **g**. Table with Hazard Ratios (HR) and 95% confidence intervals (CI, between brackets) for MFS of immune cell densities (significant HR values are shown in bold, analyses not corrected for multiple testing). Cohort A was used for panels **a-g**, n=68 LNN TNBC of which n=20 excluded, n=22 ignored and n=26 inflamed). Significant differences

are: ***, p<0.001; **, p<0.01; *, p<0.05, NS, p>0.5 (Kruskal-Wallis, one-sided). Source data are provided within source data file.

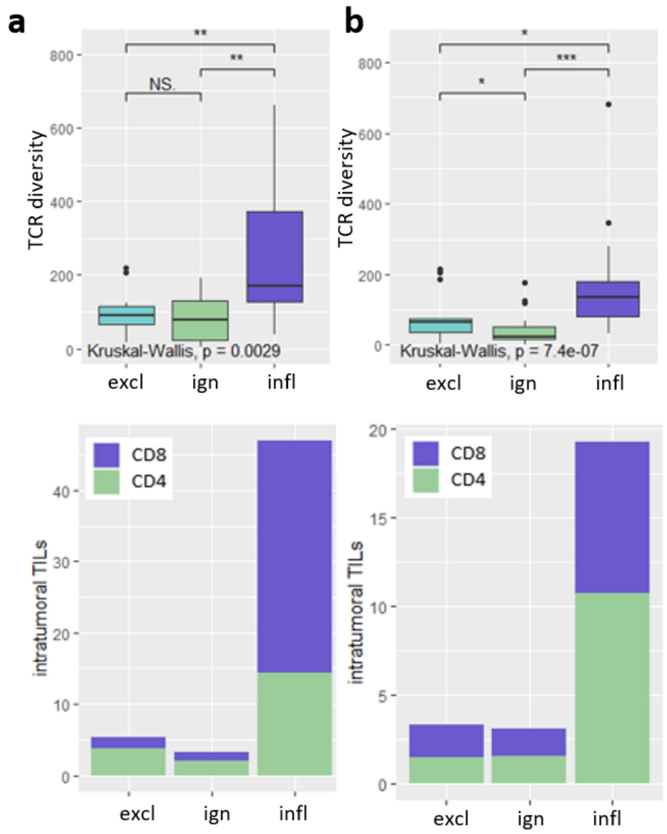

Supplementary Fig. 12. Spatial phenotypes according to CD8 staining and gene classifier are non-different with respect to TCR repertoire and presence of TILs. a and **b.** Boxplots display median with 25th-75th percentile and range (outliers are displayed as dots) for diversity of TCR-Vβ read counts (top) and intra-tumoral T-cells (cells/mm^2, bottom) of spatial phenotypes that were either based on CD8+ T-cell stainings (**a**, Cohort A2: n=43 TNBC of which n=14 excluded, n=13 ignored and n=16 inflamed) or gene classifier (**b**, Cohort C: n=66 TNBC of which n=13 excluded, n=29 ignored and n=24 inflamed). Source data are provided within source data file.

References

1. Henriksen, A., Dyhl-Polk, A., Chen, I. & Nielsen, D. Checkpoint inhibitors in pancreatic cancer. *Cancer Treat. Rev.* **78**, 17–30 (2019).

2. Kwa, M. J. & Adams, S. Checkpoint inhibitors in triple-negative breast cancer (TNBC): Where to go from here. *Cancer* **124**, 2086–2103 (2018).

3. Fay, A. P. & Antonarakis, E. S. Blocking the PD-1/PD-L1 axis in advanced prostate cancer: Are we moving in the right direction? *J. Threat. Taxa* **7**, 2–5 (2019).

4. Oliva, M. *et al.* Immune biomarkers of response to immune-checkpoint inhibitors in head and neck squamous cell carcinoma. *Ann. Oncol.* **30**, 57–67 (2019).

5. Kamatham, S., Shahjehan, F. & Kasi, P. M. Immune Checkpoint Inhibitors in Metastatic Colorectal Cancer: Current Status, Recent Advances, and Future Directions. *Curr. Colorectal Cancer Rep.* **15**, 112–121 (2019).

6. Kim, H. S. & Seo, H. K. Immune checkpoint inhibitors for urothelial carcinoma. *Investig. Clin. Urol.* **59**, 285–296 (2018).

7. Regzedmaa, O., Zhang, H., Liu, H. & Chen, J. Immune checkpoint inhibitors for small cell lung cancer: Opportunities and challenges. *Onco. Targets. Ther.* **12**, 4605–4620 (2019).

8. Liu, Y. *et al.* PD-1/PD-L1 inhibitors in cervical cancer. *Front. Pharmacol.* **10**, 1–8 (2019).

9. Flynn, J. P., O'Hara, M. H. & Gandhi, S. J. Preclinical rationale for combining radiation therapy and immunotherapy beyond checkpoint inhibitors (i.e., CART). *Transl. Lung Cancer Res.* **6**, 159–168 (2007).

10. Ayers, M. *et al.* IFN- γ – related mRNA profile predicts clinical response to PD-1 blockade. *J. Clin. Invest.* **127**, 1–11 (2017).

11. Jerby-Arnon, L. *et al.* A Cancer Cell Program Promotes T Cell Exclusion and Resistance to Checkpoint Blockade. *Cell* **175**, 984–997.e24 (2018).

12. Cabrita, R. *et al.* Tertiary lymphoid structures improve immunotherapy and survival in melanoma. *Nature* **577**, (2020).
