# Supplementary material for: Spatial immunophenotypes predict response to anti-PD1 treatment and capture distinct paths of T cell evasion in triple negative breast cancer
Source: Nat Commun. 2021 Sep 27;12:5668. doi: 10.1038/s41467-021-25962-0 (PMC8476574; doi:10.1038/s41467-021-25962-0)
Supplement: Supplementary file 2 — Reporting Summary [file 41467_2021_25962_MOESM2_ESM.pdf]

nature research

Corresponding author(s): Reno Debets

Last updated by author(s): Jul 15, 2021

Reporting Summary

Nature Research wishes to improve the reproducibility of the work that we publish. This form provides structure for consistency and transparency in reporting. For further information on Nature Research policies, see our Editorial Policies and the Editorial Policy Checklist.

Statistics

For all statistical analyses, confirm that the following items are present in the figure legend, table legend, main text, or Methods section.

n/a	Confirmed	
☐	☒	The exact sample size (*n*) for each experimental group/condition, given as a discrete number and unit of measurement
☐	☒	A statement on whether measurements were taken from distinct samples or whether the same sample was measured repeatedly
☐	☒	The statistical test(s) used AND whether they are one- or two-sided<br>*Only common tests should be described solely by name; describe more complex techniques in the Methods section.*
☐	☒	A description of all covariates tested
☐	☒	A description of any assumptions or corrections, such as tests of normality and adjustment for multiple comparisons
☐	☒	A full description of the statistical parameters including central tendency (e.g. means) or other basic estimates (e.g. regression coefficient) AND variation (e.g. standard deviation) or associated estimates of uncertainty (e.g. confidence intervals)
☐	☒	For null hypothesis testing, the test statistic (e.g. *F*, *t*, *r*) with confidence intervals, effect sizes, degrees of freedom and *P* value noted<br>*Give P values as exact values whenever suitable.*
☒	☐	For Bayesian analysis, information on the choice of priors and Markov chain Monte Carlo settings
☒	☐	For hierarchical and complex designs, identification of the appropriate level for tests and full reporting of outcomes
☐	☒	Estimates of effect sizes (e.g. Cohen's *d*, Pearson's *r*), indicating how they were calculated

Our web collection on statistics for biologists contains articles on many of the points above.

Software and code

Policy information about availability of computer code

Data collection	Imaging data was collected and analyzed using Vectra 3.0 and Inform software version 2.3. RNAseq data was collected from fresh frozen triple negative breast cancer (TNBC) using 150 bp paired-end with LncRNA library (Ribo-zero RNA) on Illumina HiSeq.
Data analysis	Data was processed in Phyton version 3.7 and R Studio version 3.5.1.

For manuscripts utilizing custom algorithms or software that are central to the research but not yet described in published literature, software must be made available to editors and reviewers. We strongly encourage code deposition in a community repository (e.g. GitHub). See the Nature Research guidelines for submitting code & software for further information.

Data

Policy information about availability of data

All manuscripts must include a data availability statement. This statement should provide the following information, where applicable:

- Accession codes, unique identifiers, or web links for publicly available datasets
- A list of figures that have associated raw data
- A description of any restrictions on data availability

All WGS, RNAseq and microarray data are available at the Gene-Expression Omnibus (GEO), European-Genome Phenome Archive and USCS Xena browser: RNAseq data from Cohort A is available through at GEO (GSE177043 https://www.ncbi.nlm.nih.gov/geo/query/acc.cgi?acc=GSE177043). Microarray data from Cohort A is available at GEO (see Supplementary File 2 for individual sample accession codes). For processed imaging and immunogenomix data of Cohort A see Supplementary File 2. Microarray data from Cohort B is available at GEO (GSE2034 https://www.ncbi.nlm.nih.gov/geo/query/acc.cgi?acc=gse2034 , GSE5327 https://www.ncbi.nlm.nih.gov/geo/query/acc.cgi?acc=gse5327 , GSE11121 https://www.ncbi.nlm.nih.gov/geo/query/acc.cgi?acc=GSE2990 , GSE2990 https://www.ncbi.nlm.nih.gov/geo/query/acc.cgi?acc=GSE11121 and GSE7390 https://www.ncbi.nlm.nih.gov/geo/query/acc.cgi?acc=gse7390). RNAseq data from Cohort

C is available at EGA (EGAS00001001178, https://ega-archive.org/studies/EGAS00001001178). RNAseq data from Cohort D is available at EGA (EGAS00001003535, https://ega-archive.org/studies/EGAS00001003535). Cohort E has been retrieved from https://xenabrowser.net/ (TCGA), And from GEO (GSE78220 https://www.ncbi.nlm.nih.gov/geo/query/acc.cgi?acc=GSE78220 , GSE91061 https://www.ncbi.nlm.nih.gov/geo/query/acc.cgi?acc=GSE91061). Classifier gene expressions and IHC scores from Cohort F are provided in Supplementary File 3. All other code and data will be made available upon request.

Field-specific reporting

Please select the one below that is the best fit for your research. If you are not sure, read the appropriate sections before making your selection.

- [x] Life sciences
- [ ] Behavioural & social sciences
- [ ] Ecological, evolutionary & environmental sciences

For a reference copy of the document with all sections, see nature.com/documents/nr-reporting-summary-flat.pdf

Life sciences study design

All studies must disclose on these points even when the disclosure is negative.

Sample size	sample size for assessment of prognostic value of spatial immunophenotypes was determined using power calculations, based on initial results of a pilot study.
Data exclusions	no data was excluded, except for samples that did not meet pre-established inclusion criteria. I.e. RNAseq samples derived from FFPE tissue that did not meet quality requirements (Cohort F) and samples that could not be assigned using the spatial phenotype gene-classifier due to equal scores for 2 phenotypes were excluded for further analysis.
Replication	No repeated measurements were performed for individual samples. The spatial phenotype classifier was validated using independent TNBC samples (n=43) ; prognostic value of the classifier was validated in 2 independent datasets (n=196 and n=137 TNBC), and the predictive value was determined using a data set of anti-PD1 treated TNBC (n=53). No inconsistencies were observed. Each immunofluorescence staining panel was performed on n>30 samples.
Randomization	For the initial discovery of spatial immunophenotypes via immune stainings we used a random set of 122 node-negative, untreated TNBC. for in depth analyses using multiplexed immune stainings we randomly selected n=25 tumors of each spatial phenotype of the above set. no selection was done for cohorts with gene-expression data.
Blinding	All data collection, genomics analysis as well as histological scoring and image analysis was performed blinded.

Reporting for specific materials, systems and methods

We require information from authors about some types of materials, experimental systems and methods used in many studies. Here, indicate whether each material, system or method listed is relevant to your study. If you are not sure if a list item applies to your research, read the appropriate section before selecting a response.

Materials & experimental systems

n/a	Involved in the study	
[ ]	[x]	Antibodies
[x]	[ ]	Eukaryotic cell lines
[x]	[ ]	Palaeontology and archaeology
[x]	[ ]	Animals and other organisms
[ ]	[x]	Human research participants
[ ]	[x]	Clinical data
[x]	[ ]	Dual use research of concern

Methods

n/a	Involved in the study	
[x]	[ ]	ChIP-seq
[x]	[ ]	Flow cytometry
[x]	[ ]	MRI-based neuroimaging

Antibodies

Antibodies used	IHC: CD8 (C8/C144B, Sanio, 1:100, pH 9); CD3 (PS1, Sigma, 1:25, pH 6); CD4 (4B12, DAKO, 1:80, pH 9), CD137 (BBK-2, Santa Cruz, 1:80, pH 6), CD278 (SP98, Thermo Fisher, 1:50, pH 9), CD66b (80H3, BIO-RAD, 1:100, pH 9), MECA-79 (C111-6, Santa Cruz, 1:50, pH 9), and MHC-II (LN3, Thermo Fisher, 1:50, pH 9);<br>IF: CD56 (MRQ-42, Sanbio, 1:500); CD3 (SP7, Sigma, 1:350), CD20 (L26, Sanbio, 1:1000); CD8 (C8/144b, Sanbio, 1:250), CD68 (KP-1, Sanbio, 1:250), Cytokeratin-Pan (AE1/AE3, Thermofisher, 1:200), CLEC9A (sheep polyclonal*, R&D Systems, 1:600) , S100A7 (47C1068, Biotechne, 1:1000), CD11b (EP1345Y, Abcam, 1:200), CD163 (MRQ26, Cell Marque, 1:50), COL10A1 (X53, Life Technologies, 1:50), Sheep IgG VisUCyte HRP polymer (R&D systems, prediluted formulation).
Validation	All immune cell markers (CD8, CD3, CD4, CD137, CD278, CD66b, MECA-79, MHC-II, CD56, CD20, CD68, CD163, CLEC9A, CD11b) were validated on human lymph node or tonsil FFPE-sections as well as inflamed TNBC FFPE-sections. Non-immune cell markers that are specific for spatial immunophenotypes (S100A7, COL10A1) were validated in TNBC samples with high and low mRNA expression of

the respective marker. Pan-cytokeratin was validated in FFPE sections of different tumor and normal tissue FFPE sections. To this end, FFPE slides were deparrafinized, and staining with primary antibodies was performed following heat induced antigen retrieval at either pH6 or pH9 (or both, in case not further specified by the manufacturer) and visialized whith HRP-polymer and opal fluorophores (Akoya). Each antibody was titrated to determine the optimal concentration.

Human research participants

Policy information about studies involving human research participants

Population characteristics	Cohort A consisted of 228 untreated, FFPE tumor specimen. All patients were female and the mean age was 52.<br><br>Clinicopathological characteristics:<br>BC subtype: 100% TNBC (ER-negative, PR-negative, Her2-negative);<br>Tumor size: 33% T1; 47% T2; 4% T3; 1% T4; 15% NA;<br>Nodal status: 70% LN0; 16% LN+; 14% NA;<br><br>The following subsets of this cohort were used to study different aspects of spatial immunophenotypes:<br>prognosis discovery: n=122 (LNN with clinical records >10 year follow up); gene classifer discovery: n=101 (microarray); classifier validation: n=43 (RNAseq); immune effector panel: n=64; spatial phenotype panel: n=69; IHC (multiple markers): n=30<br><br>Cohort F consisted of n=12 primary TNBC (grade 2 and 3) and n=12 matched lymph node metastases from patients who received neoadjuvant chemotherapy.<br><br>For details on previously published, publicly available datasets (Cohort B-E) see respective references provided in the methods section and in supplementary table 1.
Recruitment	Cohort A and F: Samples were collected as standard of care and spare tissues were used for research. Therefor, no active recruitment has taken place. For details on previously published, publicly available datasets (Cohort B-E) see respective references provided in methods section and in supplementary table 1.
Ethics oversight	Cohort A and F: The use of clinical data and spare tumor tissue for immune stainings and transcriptome analysis was approved by the medical ethics committe of erasmus MC (MEC.02.953, MEC-2020-0090). For details on previously published, publicly available datasets see respective references provided in methods and supplementary table 1.

Note that full information on the approval of the study protocol must also be provided in the manuscript.

Clinical data

Policy information about clinical studies

All manuscripts should comply with the ICMJEguidelines for publication of clinical research and a completedCONSORT checklist must be included with all submissions.

Clinical trial registration	not applicable for Cohort A and F; For cohort D see TONIC trial (NCT02499367)
Study protocol	For details on study protocol of the TONIC trial see reference 8.
Data collection	Clinical data of the TONIC trial was collected at the Netherlands Cancer Institutebetween 2015-2017. Clinical data for Cohort A were collected at Erasmus MC from 1985 onwards. For details on previously published, publicly available datasets (Cohort B-E) see respective references provided in methods and supplementary table 1.
Outcomes	clinical records included: overall survival, disease free survival, distant metastasis survival, location of metastasis, and clinicopathological characteristics such as histological subtypes, tumor grade, tumor size, metastatic activity (Cohort A)<br><br>Outcomes of the study:<br>-discovery of spatial immunophenotypes based on CD8 immune stainings on whole tumor sections (Cohort A)<br><br>-discovery and validation of the spatial immunophenotype gene-classifier (Cohort A)<br><br>-prognostic value of spatial immunophenotypes (Cohort A-D<br><br>-predictive value of spatial immunophenotype gene-classifier in cohort E (publicly available anti-PD1 treated TNBC)<br><br>-drivers and evasive mechanims of spatial immunophenotypes (i.e.; relation to clinical data, genomic features transcriptome data and immunological data; Cohort A and Cohort C)
