# Supplementary material for: Spatial immunophenotypes predict response to anti-PD1 treatment and capture distinct paths of T cell evasion in triple negative breast cancer
Source: Nat Commun. 2021 Sep 27;12:5668. doi: 10.1038/s41467-021-25962-0 (PMC8476574; doi:10.1038/s41467-021-25962-0)
Supplement: Supplementary file 7 — Description of Additional Supplementary Files [file 41467_2021_25962_MOESM7_ESM.pdf]

Title: Supplementary Data 1.
Description: Classifier genes with Affymetrix ID, HGNC Gene Symbol, ENTREZ ID and expression rank in excluded, ignored and inflamed triple-negative breast cancer.

Title: Supplementary Data 2.
Description: General information regarding data of Cohort A; individual GSM accession codes and coded sample annotation that allow for integrative analyses of clinical-, imaging- and omics data; extended clinical data; extended pathological and IHC scores including T cell density, necrosis and tertiary lymphoid structures (TLS); omics analyses including MIXCR and CIBERSORT outputs; imaging of individual immune markers (manually scored as percentage of all cells) as well as multispectral imaging data regarding immune effector cells and markers related to T cell evasion derived through VECTRA/inform analysis (numbers of cells per mm^2 and collagen-10 as area).

Title: Supplementary Data 3.
Description: Immunophenotypes based on IHC and classifier gene expressions (raw counts and gene lengths) of cohort F which includes lymph node metastases and matched primary triple negative breast cancer.
