# Supplementary material for: Spatial immunophenotypes predict response to anti-PD1 treatment and capture distinct paths of T cell evasion in triple negative breast cancer
Source: Nat Commun. 2021 Sep 27;12:5668. doi: 10.1038/s41467-021-25962-0 (PMC8476574; doi:10.1038/s41467-021-25962-0)
Supplement: Supplementary file 6 — Peer Review File [file 41467_2021_25962_MOESM6_ESM.pdf]

Reviewers' Comments:

Reviewer #1:
Remarks to the Author:
In this report, Hammerl and colleagues investigated the spatial contexture of CD8+ T cells in a TNBC cohort and identified T-cell inflamed, excluded and ignored subtypes with prognostic implications. A subset of these tumors were expression profiled, and subtype-discriminatory genes were identified from which a T-cell subtype gene-based classifier was constructed. The classifier performed with reasonable accuracy (though could not discriminate excluded from ignored subtypes) and consistently retained prognostic value in independent breast tumor cohorts. In a phase II trial of anti-PD1 in metastatic TNBC, the gene classifier significantly discerned responders and nonresponders, outperforming other predictive measures including TMB and PD-L1 staining. Further, gene and cellular differences between T-cell subtypes were identified, implicating immunostimulatory and immunosuppressive/evasive pathways in ICI outcomes.

Strengths:
- This study represents the first attempt in breast cancer to reconcile tumor T-cell spatial architecture with gene expression signatures predictive of immune checkpoint blockade response in breast cancer patients.
- The study confirms the prognostic and treatment-predictive power of T-cell-inflamed genes previously observed in breast and other cancer types.
- The study points to possible new molecular targets in TNBC, such as collagen-10 deposition and CLEC9A+ dendritic cells, that if therapeutically manipulated could enhance ICI outcomes.

Concerns:
- The logic in transitioning from IHC-based T-cell spatial subtyping to a gene-based signature is unclear. Since the signature shows some classification inaccuracy, might the IHC-based spatial subtyping approach result in better classification of anti-PD1 responders and nonresponders?
- The clinical relevance of the gene classifier is uncertain. Do multivariable Cox or logistic regression models that include other prognostic variables such as patient age, tumor size, histologic grade, LN status, stage, etc, confirm that the prognostic value of the gene signature is independent of these variables?
- Tertiary lymphoid structures (TLS) were identified in both inflamed and excluded subtypes. TLS are associated with improved patient outcomes and activation of anti-tumor immunity. Are TLS independently associated with patient survival within the excluded/ignored and inflamed subtypes? If so, can these structures also be “predicted” by a gene signature and incorporated into the current classification system for improved prognostic/predictive performance?
- How gene-based classifiers are constructed can impact their ultimate performance. Cutoffs used for DE gene selection are not clear. How the classifier was trained is not clear – was a form of cross-validation used to guard against overfitting? Such details are needed for other researchers to reproduce the work.
- While the biological characterization of the subtypes revealed novel insights and potential new targets, these findings are thus far correlative in nature. Demonstration of functional roles for one or more of the named variables, such as collagen-10 overexpression, in promoting T cell exclusion would increase scientific merit.
- Many immune gene classifiers capable of discerning short and long survival, as well as treatment responses, have been described for breast cancer and other cancer types, including ICI treated cancer cohorts. What are the value-added aspects of your gene signature? Does your signature outperform other published signatures?

Reviewer #2:
Remarks to the Author:
Major issues:
1. More clearly define ‘stromal’ and ‘intra-tumoral’ – are these mutually exclusive? Is stromal only at tumor border or also within tumor center?
2. Much of their results were already published in Gruosso et al, JCI 2019 (citation #18 in this manuscript).

3. In the Gruosso 2019 JCI paper, they further divided inflamed TNBC tumors into stromal accumulation vs. epithelial infiltration (analogous to tumor cell clusters, citation #53 in this manuscript). Authors should also do this.
4. Authors overstated their results in metastasis and response to anti-PD1 therapy. Spatial immunophenotypes were done in primary tumors, not mets, and do not directly predict anti-PD1 response. They derived a gene expression signature from primary tumors based on spatial immunophenotypes, then applied this gene signature to metastatic samples in relation to anti-PD1 responses.
5. Cohort D is the only metastatic samples. It is possible/likely that gene signatures of metastatic samples may be different from primary tumors. As such, it would be important to do similar IHC analysis of at least some met samples in cohort D to confirm their spatial patterns.

Reviewer #3:
Remarks to the Author:
GENERAL IMPRESSION:

This is an interesting paper describing novel prognostic value for immune spatial phenotypes in TNBC. The authors use multiple independent cohorts and their findings are clearly presented, tying everything in a scientific narrative that makes sense, is easy to understand, and whose direct clinical implications are clearly presented. The paper is well written, and presents a significant addition to the literature on the topic, with clear directions for future research.

MAJOR COMMENTS:

1. While the authors report the results for genomic subsets, i.e. TNBC, they do not report the results for histological subsets. Of particular relevance are infiltrating lobular carcinomas where the concepts of "tumor border" are hard to define. How do the author's conclusions hold for histological phenotypes where there is no definite tumor boundary? This question does not only apply to breast cancer, but to other tumor sites as well.

2. The authors use a gene expression classifier as a proxy for visual examination of IHC stained slides to characterize spatial immune contexture in cohorts where IHC is not available. The authors should make it clear that this is an indirect relationship, and relies on correlations that may not necessarily hold for different patient subsets and tumor sites. The authors do briefly mention this fact in the limitations, but I recommend expanding on this, and using a more conservative language when interpreting the findings that rely exclusively on the gene expression classifier, such those done in non-breast-cancer cohorts from the TCGA.

3. The authors use a very simple differential gene expression analysis framework, even though there are state-of-the-art methods exist. The limitations of this simple approach are best described in the introduction section of the seminal paper by Subramanian et al, 2005, "Gene set enrichment analysis: A knowledge-based approach for interpreting genome-wide expression profiles". Proceedings of the National Academy of Sciences. 102 (43): 15545–15550:

"

A common approach involves focusing on a handful of genes at the top and bottom of L (i.e., those showing the largest difference) to discern telltale biological clues. This approach has a few major limitations.

(i) After correcting for multiple hypotheses testing, no individual gene may meet the threshold for statistical significance, because the relevant biological differences are modest relative to the noise inherent to the microarray technology.

(ii) Alternatively, one may be left with a long list of statistically significant genes without any unifying biological theme. Interpretation can be daunting and ad hoc, being dependent on a

biologist’s area of expertise.

(iii) Single-gene analysis may miss important effects on pathways. Cellular processes often affect sets of genes acting in concert. An increase of 20% in all genes encoding members of a metabolic pathway may dramatically alter the flux through the pathway and may be more important than a 20-fold increase in a single gene.

(iv) When different groups study the same biological system, the list of statistically significant genes from the two studies may show distressingly little overlap (3)

"

To be clear, I am not necessarily recommending that the authors use GSEA specifically, but some sort of "second generation"/"Functional Class Scoring" pathway analysis (including GSEA or recent improvements on it) is probably the better approach to use than simple differential expression and averaging. Please take a look at the following review by Butte et al for reference: Khatri P, Sirota M, Butte AJ (2012) Ten Years of Pathway Analysis: Current Approaches and Outstanding Challenges. PLOS Computational Biology 8(2): e1002375. https://doi.org/10.1371/journal.pcbi.1002375

MINOR COMMENTS:

1. Figure 2F is inadequately explained in the figure legend of the corresponding text. What is the predictor here? Do the various panels show predictions for ignored-vs-others, excluded-vs-others, and ignored/excluded-vs-inflamed in predicting response to ICI? I recommend clarifying the text here.

2. A brief discussion on the limitations of visual assessment (ambiguity, non-representativeness of examined regions compared to full tumor, etc) is needed. The authors should explain in the limitations that future studies, wherever practically possible, can use quantitative computational approaches instead for this task.

3. Likewise, a brief discussion of the limitations of digital image analysis is needed. For example, the authors state in the methods that "Tissue-segmentation, cell-segmentation

and phenotyping of individual cells was performed using Inform software". While commercial software tends to do a reasonable job for many tasks, it should be noted that misclassifications and errors in segmentation are not uncommon. The authors should mention this somewhere in the limitations as a confounder to the analysis.

Response to Reviewers' comments to original manuscript NCOMMS-20-22117

Reviewer#1

Comment 1:
The logic in transitioning from IHC-based T-cell spatial subtyping to a gene-based signature is unclear. Since the signature shows some classification inaccuracy, might the IHC-based spatial subtyping approach result in better classification of anti-PD1 responders and nonresponders?

Response:
Authors thank R1 for requesting clarification regarding the transitioning from IHC to gene-based spatial T cell phenotyping. The rationale for designing, validating and utilizing a gene-classifier rather than IHC to classify spatial T cell phenotypes is that whole tissue sections, in particular for metastasized tumors such as from anti-PD1 treated patients, are not standardly available for diagnosis and in particular for research. For such patients, mostly small biopsies consisting of fragmented tissue parts are available, which are not generally adequate for spatial T cell phenotypes (i.e., lack of tumor borders does not allow accurate distinction between ignored and excluded phenotypes). In case of gene-based classification, one would require tissue-derived RNA expressions from those genes that are part of our proposed signature which can be determined via standard molecular techniques and developed into a diagnostic tool. Alternatively, NGS-techniques are expected to be implemented at Pathology departments of Medical Centers to become part of systemic evaluation of targetable alterations in the near future.

The above arguments, and also not ruling out IHC-based classifications for routine purposes, can be found in the revised Discussion section (see **line# 302-327,** highlighted in yellow).

Comment 2:
The clinical relevance of the gene classifier is uncertain. Do multivariable Cox or logistic regression models that include other prognostic variables such as patient age, tumor size, histologic grade, LN status, stage, etc, confirm that the prognostic value of the gene signature is independent of these variables?

Response:
We commend R1 for suggesting to test the confounding effect of other prognostic features regarding an association between spatial immunophenotypes and clinical outcome. To this end, we have performed multivariable analysis for spatial phenotypes based on IHC (cohort A) as well as gene expression (cohort E) using the clinical parameters age, tumor size, tumor grade and nodal status. Of note, nodal status was not included for cohort A since all patients used for survival analysis were lymph node-negative.

Our analysis revealed that using the multivariable model spatial immunophenotypes based on IHC or gene expression remained significantly associated with overall survival (OS) ($p \leq 0.009$, see **Table 1A** and **1B**). Similar associations were found for metastasis-free survival and disease-free survival ($p \leq 0.009$). The lack of prognostic significance of histological grade in TNBC is consistent with a previously published pooled analysis of 9 Phase 3 adjuvant TNBC-trials (Loi, J Clin Oncol 2019). We conclude that spatial immunophenotype in comparison to age, tumor size, tumor grade and nodal status proves to be the only consistent prognosticator. This is now mentioned in the Results section (**line #109-111**) and below tables are added to the supplementary data (**Supplementary Table S2**) of the revised manuscript.

A Covariate	HR (CI)	p-value
immunophenotype based on IHC	0.31 (0.13-0.75)	0.009
tumor size	1.01 (0.98-1.05)	0.19
grade	0.89 (0.49-1.6)	0.69
age	0.98 (0.95-1.01)	0.13

B Covariate	HR (CI)	p-value
immunophenotype based on gene expression	0.17 (0.05-0.58)	0.004
nodal status	6.83 (0.85-54.8)	0.07
tumor size	1.17 (0.4-3.39)	0.76
age	1.02 (0.98-1.05)	0.23

Table 1. A. Multivariable Cox regression analysis including spatial immunophenotypes based on IHC (Cohort A, n=106). **B**. Multivariable Cox regression analysis including spatial immunophenotypes based on gene-expression (Cohort E, n=140). All spatial immunophenotypes were part of analysis, but outcomes are displayed for inflamed immunophenotype. Abbreviations: HR: hazard ratio, CI: confidence interval.

Comment 3:

Tertiary lymphoid structures (TLS) were identified in both inflamed and excluded subtypes. TLS are associated with improved patient outcomes and activation of anti-tumor immunity. Are TLS independently associated with patient survival within the excluded/ignored and inflamed subtypes? If so, can these structures also be "predicted" by a gene signature and incorporated into the current classification system for improved prognostic/predictive performance?

Response:

In line with R1's suggestion we have evaluated the prognostic value of the presence and frequency of TLS per spatial immunophenotype. TLS, when scored as dense clusters of CD4+ T-cells and CD20+ B cells (as described in the Materials and Methods section), did not significantly associate with survival outcomes in a univariate model (**Figure 1A)**. Overall survival, but not metastasis-free survival nor disease-free survival, showed a trend towards association with TLS (OS: HR 0.54 (CI 0.3-1.02) p=0.05); MFS: HR 0.81 (CI 0.4-1.6) p=0.18); DFS: HR 1.08 (CI 0.56-2) p=0.63). Using the same set of patients, all three survival outcomes were clearly associated with spatial immunophenotypes, and the association between phenotypes and OS was not improved when stratifying for TLS (**Figure 1B, C**). Importantly, using a multivariate model, TLS did not significantly associate with any of the three survival outcomes, whereas spatial immunophenotypes did significantly associate with all three survival outcomes (**Figure 1C, Table 2A**).

In addition to immune staining, we also scored TLS using a gene expression signature with reported prognostic and predictive value in melanoma (Cabrita, Nature 2020). Using this signature, we again could not demonstrate a significant association with OS whether it be in univariate (HR 0.88 (CI 0.52-1.5) p=0.65) or multivariable settings (**Table 2B**). Notably, this

signature was also not associated with response to anti-PD1 in multivariable analysis (**Table 2C**, and for details see response to **R1's comment 6**).

In conclusion, and based on our datasets, TLS is not of added value for the prognosis or prediction of anti-PD1 response according to spatial immunophenotypes in TNBC. These findings do warrant further research into the exact role of TLS in shaping anti-tumor immune responses in TNBC, particularly its biological relationship to spatial immunophenotypes. We have included above findings and interpretations in the revised Results section (**line# 130-132**), **Table S2** and Discussion section (**line# 311-315**).

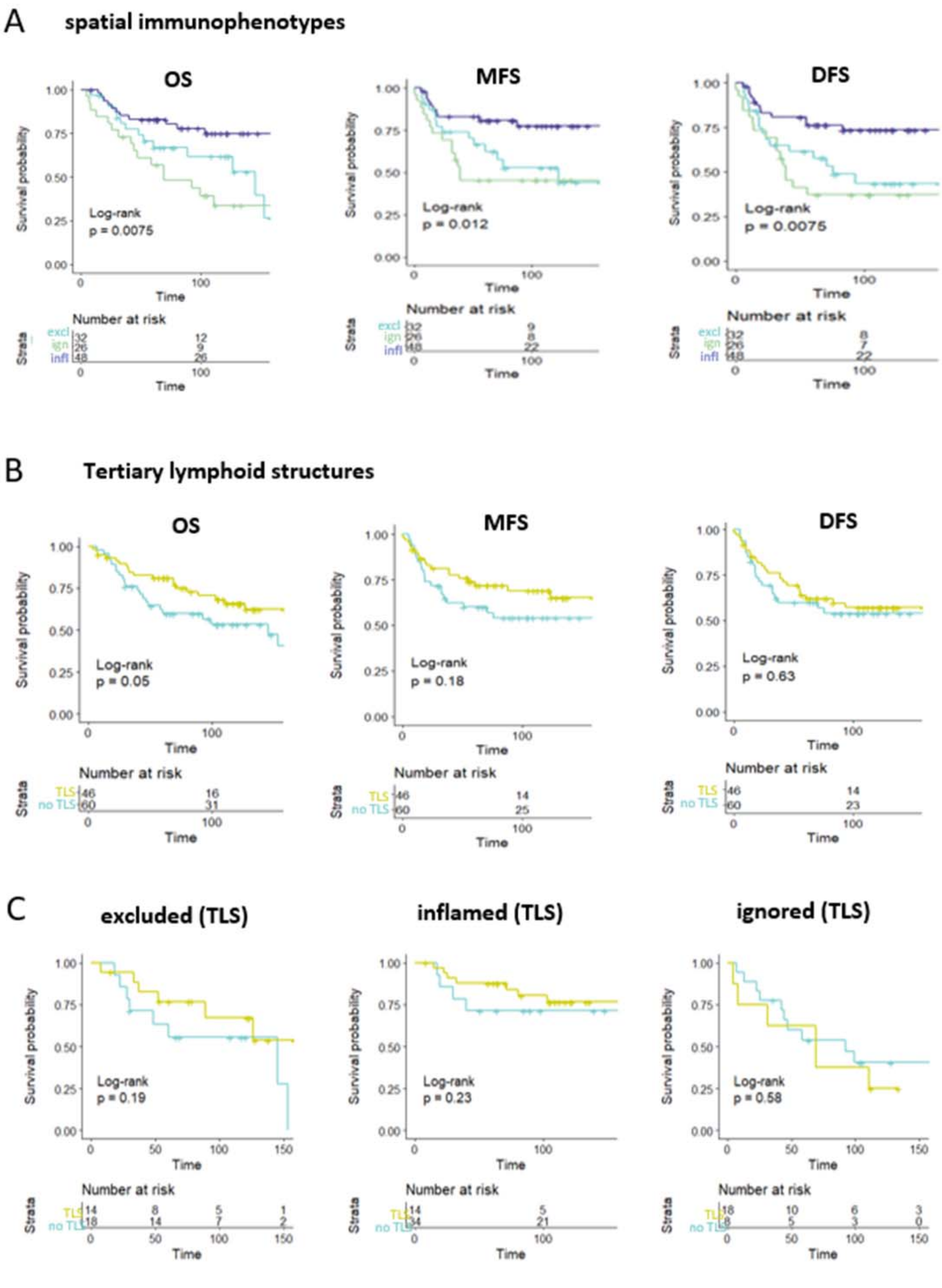

Figure 1. Prognostic value of tertiary lymphoid structures (TLS) stratified per spatial immunophenotype in TNBC. A. Kaplan-Meier curves show overall survival (OS), metastasis-free survival (MFS) and disease-free survival (DFS) according to spatial immunophenotype. **B.** Kaplan-meier curves show OS, MFS and DFS according to presence of TLS. **C.** Kaplan-Meier curves show OS for the three spatial immunophenotypes in Cohort A stratified for the presence of TLS. (Cohort A, n=106 LNN primary TNBC)

A	Covariate	OS: HR (CI) p-value	MFS: HR (CI) p-value	DFS: HR (CI) p-value
**spatial immunophenotype based on IHC**		0.31 (0.13-0.75) p=0.009	0.32 (0.13-0.78) p=0.01	0.28 (0.12-0.65) p=0.003
**TLS presence**		0.64 (0.29-1.5) p=0.28	0.75 (0.31-1.77) p=0.51	1.09 (0.5-2.4) p=0.81
**TLS frequency**		1.00 (0.78-1.17) p=0.13	1.06 (0.88-1.2) p=0.5	1.04 (0.89-1.23) p=0.59

B	Covariate	HR (CI) p-value
**spatial immunophenotype based on gene expression**		0.17 (0.05-0.51) p=0.002
**TLS signature (Carbrita, Nature, 2020)**		1.3 (0.7-2.5) p=0.38

C	Covariate	OR (CI) p-value
**spatial immunophenotype based on gene expression**		7.25 (1.39-47.13) p=0.02
**TLS signature (Carbrita, Nature, 2020)**		0.99 (0.46-2.1) p=0.99

Table 2. Multivariable survival analysis of spatial immunophenotypes and TLS. A. Spatial immunophenotypes and TLS based on IHC (Cohort A, n=106 LNN primary TNBC). **B**. Multivariable overall survival analysis including TLS based on gene expression (Cohort E, n=145 primary TNBC). **C.** Multivariable analysis for response to anti-PD1 including TLS based on gene expression (Cohort D, n=51 metastatic TNBC). All spatial immunophenotypes were part of analysis, but outcomes are displayed for inflamed immunophenotype. Abbreviations: OS: overall survival, DFS: disease-free survival, MFS: metastasis -free survival, HR: hazard ratio, CI: confidence interval.

Comment 4:

How gene-based classifiers are constructed can impact their ultimate performance. Cutoffs used for DE gene selection are not clear. How the classifier was trained is not clear – was a form of cross-validation used to guard against overfitting? Such details are needed for other researchers to reproduce the work.

Response:

We thank R1 for providing the opportunity to better explain the construction of the gene classifier. Construction of our classifier has been performed according to top differentially expressed genes from cohort A1 (microarray data), rank correlations of classifier gene expressions and assignment based on correlation coefficients. The performance of the classifier was validated in an independent dataset primary TNBC (IHC and RNAseq data) as well as a new set of TN lymph node metastases (IHC and RNAseq data; see also response to **R2's comment 4**). This methodology was compatible to the use of different platforms (i.e., micro-array and RNAseq data), and yielded accurate classification of individual spatial immunophenotypes in at least 81% of samples in both validation sets. It is noteworthy that machine learning-based classifiers and cross-validation (i.e., the geNetClassifier package in R) did not yield such accuracy. In more detail, and along R1's specific questions, we have selected 42 genes with most discriminatory expression levels (i.e. >1logFC, p_{adj}<0.05 among all 3 spatial immunophenotypes) and did not implement machine-learning-based training nor cross validation. The Materials and Methods section of the revised manuscript has been amended accordingly (see **line# 668-691**); and all classifier genes and ranks have been added to **Supplementary File 1**, thereby enabling reproduction of our findings.

Comment 5:
While the biological characterization of the subtypes revealed novel insights and potential new targets, these findings are thus far correlative in nature. Demonstration of functional roles for one or more of the named variables, such as collagen-10 overexpression, in promoting T cell exclusion would increase scientific merit.

Response:
R1 suggests functional validation of the T cell evasive mechanisms linked to the spatial immunophenotypes in TNBC. In itself these additional experiments represent a correct and logical extension of our study outcomes. We would like to put forward, however, that the primary message of the present study entails the discovery of T cell evasive mechanisms that are differentially related to distinct spatial immunophenotypes in TNBC. These outcomes are the result of interrogating large cohorts of TNBC patients (n=699); analysis at the gene as well as spatial protein expression levels; and relating data with NGS-, immunologic- and clinical sets of patient data. Using this extensive and integrative approach, we enabled the identification of T cell evasive pathways using NGS which were subsequently validated using multiplexed immune fluorescence stainings. At this point, we are in the process to set up NSG mouse models with TNBC to test the individual targeting of such evasive mechanisms. For the current revised manuscript, given the message as it stands, as well as the timelines needed for follow-up messages, authors consider functional validations beyond its scope.

We have explicitly acknowledged the future need for functional validation of the identified T cell evasive mechanisms in TNBC; see the Discussion section; **line# 422-423**.

Comment 6:
Many immune gene classifiers capable of discerning short and long survival, as well as treatment responses, have been described for breast cancer and other cancer types, including ICI treated cancer cohorts. What are the value-added aspects of your gene signature? Does your signature outperform other published signatures?

Response:
Authors appreciate R1's valuable suggestion to compare our gene classifier to other published classifiers that predict ICI responses. Out of many reported classifiers, we have applied those that are recognized for capturing lymphocyte activity and location, being most relevant for a head-to-head comparison. These signatures included: a short (6-gene) and extended (18-gene) IFNγ-response signature that both predict anti-PD1 response in melanoma and head and neck squamous cell carcinoma (Ayers et al., JCI, 2017); a T cell exclusion signature that predicts anti-PD1 response in melanoma (Jerby-Arnon et al., Cell, 2018); and a TLS signature that predicts anti-PD1 response in melanoma (Cabrita et al., Nature, 2020). Of these signatures, the extended IFNγ signature was the only one able to predict response in a small cohort of anti-PD1-treated mTNBC patients (AUC=0.7; p=0.046), yet did not (nor any of the tested classifiers) outperform the prognostic and predictive value of our gene classifier in mTNBC (see figure below). We conclude that the spatial immunophenotype gene classifier, in contrast to other classifiers, shows clear and unprecedented prediction of response to anti-PD1 in mTNBC.

We have included below figure (as **Figure S7**) and accompanying text in the Result section (see **line# 209-212**) and Discussion section (**line# 310-315**) of the revised manuscript.

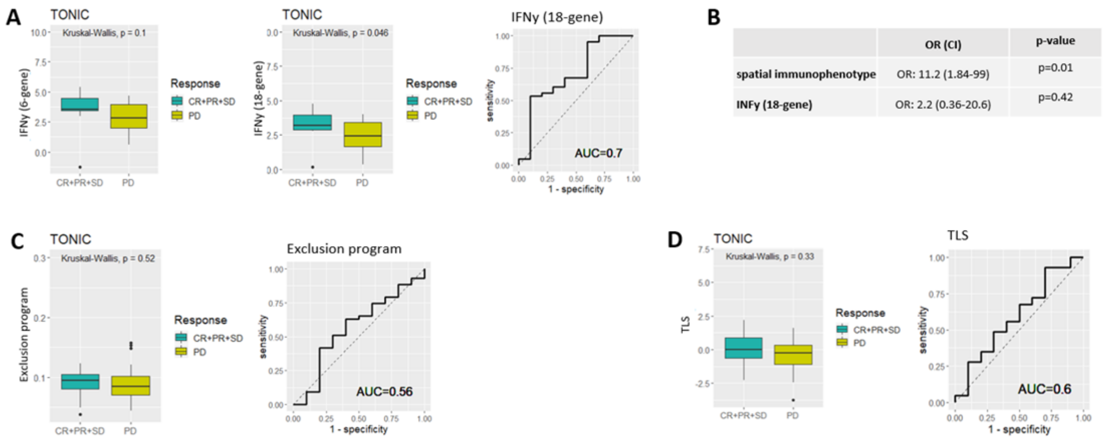

Figure 2. Predictive value of spatial immunophenotype gene classifier versus public classifiers. A. Box-plots display signature scores in responder (CR+PR+SD) and non-responder patients from TONIC trial (cohort E) (PD) according to a short (6-gene) and extended (18-gene) interferon gamma signature from Ayers et al., JCI 2017. ROC displays area under the curve for predicting anti-PD1 response for the extended signature. **B.** Multivariable analysis with spatial immunophenotype gene-classifier and the extended IFNy signature. **C**. Box plots and ROC according to a T cell exclusion program signature from Jerby-Arnon et al., Cell, 2018. **D.** Box plots and ROC according to a tertiary lymphoid structure signature from Cabrita et al., Nature, 2020.

Reviewer #2

Comment 1:

More clearly define 'stromal' and 'intra-tumoral' – are these mutually exclusive? Is stromal only at tumor border or also within tumor center?

Response:

Authors thank R2 for requesting a clear definition of stromal and intra-tumoral regions. Tumor border and center regions comprise both stromal as well as tumor cell compartments, which were defined through absence or presence of the cytokeratin marker, respectively. As illustrated in **Supplementary Figure 2B** and shown in **Supplementary Figures 3** and **11** stromal and intra-tumoral compartments are indeed mutually exclusive.

Along R2's recommendation, we have amended the description in the Materials and Methods section and referred to Supplementary Figure 2 (see **line# 594-599, 615-622**).

Comment 2:

Much of their results were already published in Gruosso et al, JCI 2019 (citation #18 in this manuscript).

Response:

We understand R2's remark, and although part of the results is overlapping, large parts of the results are novel and cover existing gaps in understanding and predicting restistance to anti-PD1 therapy in TNBC. In our study, we have used cohorts of in total 681 patients with TNBC and 4,003 patients with other cancers, and were able to present in-depth analyses of 3 spatial immunophenotypes (i.e., *ignored, excluded and inflamed*) in relation to prognosis and response to anti-PD1 treatment as well as T-cell evasion. In contrast, the study by Gruosso and colleagues have used a cohort of 38 patients with TNBC and analyzed 4 localizations of CD8 T cells (i.e., *immune desert, margin restricted, stromal restricted and fully inflamed*) in relation to prognosis.

Specifically, our study has uniquely covered:

(1) Development and validation of a gene-classifier that accurately predicts the spatial immunophenotypes in TNBC and mTNBC (also see response to **R2's comment 4**), and is associated with prognosis in 2 independent datasets of TNBC and various other cancers;
(2) Clinical validation of this gene-classifier in TNBC patients who received anti-PD1;
(3) Discovery of genomic features (i.e., numbers and types of mutations; TCR repertoire diversity; as well as clonality and mutational signatures) as well as oncogenic and immune pathways that characterize the spatial immunophenotypes (i.e., immunogenic cell death; VEGF/TGFβ signaling; WNT signaling).

In conclusion, our study provides a spatial immunophenotype gene classifier that predicts clinical response to anti-PD1 that is independent of currently used clinical markers and outperformes other gene-signatures, thereby addressing an urgent clinical need. Moreover, our in-depth analysis of NGS, immunologic and clinical sets of patient data points towards differential and actionable targets that may proof beneficial for phenotype-stratified immunotherapy in TNBC.

Comment 3:

In the Gruosso 2019 JCI paper, they further divided inflamed TNBC tumors into stromal accumulation vs. epithelial infiltration (analogous to tumor cell clusters, citation #53 in this manuscript). Authors should also do this.

Response:

The inflamed phenotype has prognostic value in our data sets (**Figure 1A**). Along R2's suggestion, we have subdivided the inflamed phenotype in TNBC into stromal restricted (SR) versus fully inflamed (FI) localization of CD8 T cells according to Gruosso et al., JCI, 2019, and observed no additional benefit to patient survival (**Figures 3A, B).** We also tested the prognostic value of the cholesterol meta-signature, which was found to be characteristic for the SR phenotype as reported by Gruosso and colleagues. Again, the subdivision into cholesterol-low versus high did not yield additional benefit either to the survival of TNBC patients with the inflamed/IFNγ hi phenotype (**Figure 3C**). Collectively, these results demonstrate that further subclassification of the inflamed phenotype in TNBC according to stromal vs intratumoral localizatioin of CD8 T cells is not of added value in our datasets. Notably, these results are in line with a recent report showing that both stromal as well as intratumoral TIL are associated with good prognosis in >1000 TNBC; that stromal and intratumoral TIL were strongly correlated and had similar prognostic value; and that inclusion of intratumoral TIL did not improve the multivariable model based on stromal TIL and clinicopahological parameters (Loi, J Clin Oncol, 2019).

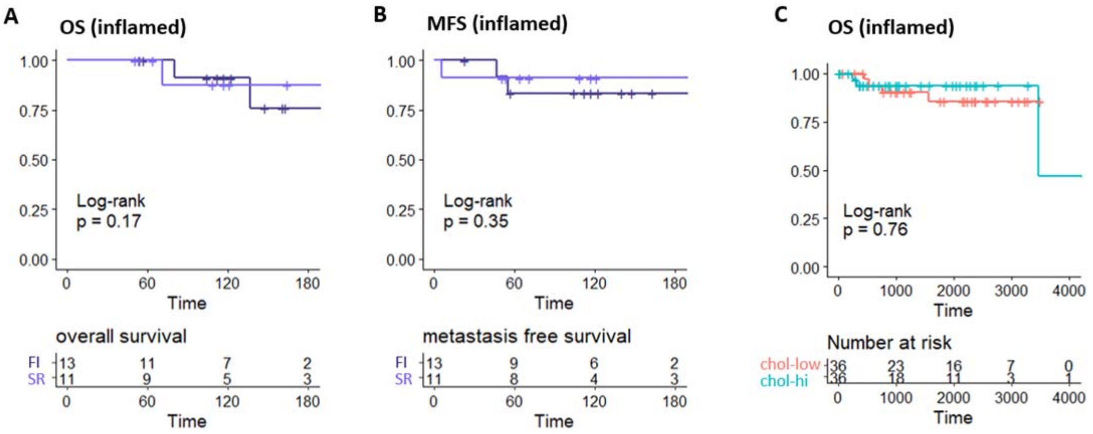

Figure 3. Subclassification of inflamed phenotype in TNBC according to localization of CD8 T cells. A,B. Kaplan Meier curves show overall survival (**A**) and metastasis-free survival (**B**) of the inflamed phenotype in TNBC stratified for stromal restricted (SR) or fully inflamed (FI) localization of CD8 T cells (according to Gruosso et al., JCI, 2019). **C**. Kaplan Meier curve shows overall survival of the inflamed phenotype in TNBC stratified for abundance of cholesterol signature (Cohort E).

Comments 4 and 5:

Authors overstated their results in metastasis and response to anti-PD1 therapy. Spatial immunophenotypes were done in primary tumors, not mets, and do not directly predict anti-PD1 response. They derived a gene expression signature from primary tumors based on spatial immunophenotypes, then applied this gene signature to metastatic samples in relation to anti-PD1 responses. Cohort D is the only metastatic samples. It is possible/likely that gene signatures of metastatic samples may be different from primary tumors. As such, it would be important to do similar IHC analysis of at least some met samples in cohort D to confirm their spatial patterns.

<u>Response:</u>

We thank R2 for this valuable recommendation, and agree that validation of our gene classifier in metastatic lesions is relevant to its clinical value. From the metastatic samples of cohort D, only biopsies are available, and consequently these samples do not allow accurate classification of spatial immunophenotypes based on immune stainings (i.e., biopsies often lack tumor border

regions thereby not enabling accurate distinction between ignored and excluded phenotypes). To overcome this limitation, we have selected a new set of LN metastases from TNBC patients that comprises FFPE-derived whole lesion sections, and performed CD8 stainings as well as RNA sequencing for these new samples. Although FFPE starting material generally yields worse quality of RNA when compared to FF samples, we still captured sequencing data of 12 out of 15 samples with sufficiently high quality: i.e., these samples contained <50% duplicated reads (range: 20-45%); >50% mapped reads (range: 55-95%); and expressed >75% of classifier genes. Classification using our spatial immunophenotype gene classifier yielded correct assignment of 10 out of 12 samples (83%, for details see **Table 3**).

These new findings extend the notion that gene-expression profiles remain rather stable between primary and metastatic breast cancer (Weigelt et al., PNAS 2003), and further substantiate that our spatial immunophenotype gene-classifier correlates with anti-PD1 response.

These new data are included as **Table 1B** and described in the Result section (**line# 144-147**) of the revised manuscript, and corresponding techniques and interpretation are described in the Materials and Methods (**line# 550, 598-599, 634-641, 682-685**) and Discussion sections (**line#303**), respectively.

B	spatial immunophenotype (gene classifier)					
CD8 stainings		excl	ign	infl	Total	sensitivity
	excl	1	1	0	2	0.50
	ign	0	3	1	4	0.75
	infl	0	0	6	6	1.00
	total	1	4	7	12	
	specificity	1.00	0.75	0.85		

Table 3. Performance of gene classifier for spatial immunophenotypes in metastatic TNBC.

Reviewer #3

Comment 1:

While the authors report the results for genomic subsets, i.e. TNBC, they do not report the results for histological subsets. Of particular relevance are infiltrating lobular carcinomas where the concepts of "tumor border" are hard to define. How do the author's conclusions hold for histological phenotypes where there is no definite tumor boundary? This question does not only apply to breast cancer, but to other tumor sites as well.

Response:

Authors thank R3 for pointing out technical challenges regarding the definition of tumor border regions in infiltrating lobular carcinoma subtypes. In our study, we observed 6 different subtypes according to histology, with invasive ductal carcinoma (IDC) representing the vast majority of TNBC. Spatial immunophenotypes according to different histological subtypes are exemplified and listed in **Supplementary Figure 8D**. Our analyses (see aforementioned figure) showed that spatial phenotypes were not associated with these subtypes, except for IDC with medullary like features, which was (expectedly) associated with the inflamed phenotype. In our dataset only 4 samples were histologically annotated as invasive lobular carcinoma (ILC) (2 inflamed, and 2 ignored phenotypes). In this study and irrespective of histological subtype, tumor border regions were defined as those areas with 50% tumor area and 50% stroma/surrounding fat/normal ducts, which, in case of ILC, may include some isolated tumor cells. Examples of ILC tumor border regions are displayed in **Figure 4** below. All samples, including the ILC samples, were assessed by experienced pathologists, and unresolved challenges regarding the definition of tumor border regions in TNBC have not been encountered.

In the revised manuscript we have specifically mentioned the staining of different histological subtypes, provided an exact definition of tumor border regions and described the implications for ILC subtypes (see **line# 563-564, 594-599**).

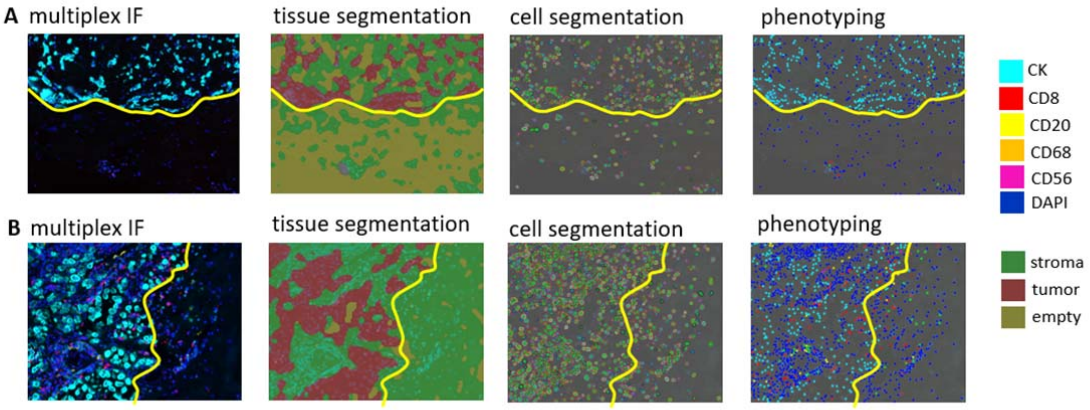

Figure 4. Image analysis of border regions of invasive locular carcinoma. A. Representative images of border region of ignored phenotype in TNBC. **B**. Representative border region of inflamed phenotype in TNBC. Yellow lines indicate tumor borders. The different steps are explained in Supplementary Figure 2. Color coding for markers as well as tissue compartments is given at right-hand side.

Comment 2:

The authors use a gene expression classifier as a proxy for visual examination of IHC stained slides to characterize spatial immune contexture in cohorts where IHC is not available. The authors should make it clear that this is an indirect relationship, and relies on correlations that may not necessarily hold for different patient subsets and tumor sites. The authors do briefly mention this fact in the limitations, but I recommend expanding on this, and using a more conservative

language when interpreting the findings that rely exclusively on the gene expression classifier, such those done in non-breast-cancer cohorts from the TCGA.

Response:

Authors commend R3 for this remark and agree that assignment of spatial phenotypes may be less accurate when using gene expression instead of immune stainings. Indeed, when substituting immune staining by gene expression in different patient subsets, tumor sites or tumor types, such as those that exclusively rely on TCGA data, there is a risk that due to lack of imaging some samples are mis-classified. Of note, in the revised manuscript, we have now included the validation of the gene classifier not only towards primary tumors, but also towards LN metastases of TNBC (see response to **R2's comment 4**). According to R3's suggestion, we have amended and expanded the text on limitations of our study, as stated above, in the Discussion section of the revised manuscript (see **line# 416-419**).

Comment 3:

The authors use a very simple differential gene expression analysis framework, even though there are state-of-the-art methods. The limitations of this simple approach are best described in the introduction section of the seminal paper by Subramanian et al, 2005, "Gene set enrichment analysis: A knowledge-based approach for interpreting genome-wide expression profiles". Proceedings of the National Academy of Sciences. 102 (43): 15545–15550"

A common approach involves focusing on a handful of genes at the top and bottom of L (i.e., those showing the largest difference) to discern tell-tale biological clues. This approach has a few major limitations.

(i) After correcting for multiple hypotheses testing, no individual gene may meet the threshold for statistical significance, because the relevant biological differences are modest relative to the noise inherent to the microarray technology.

(ii) Alternatively, one may be left with a long list of statistically significant genes without any unifying biological theme. Interpretation can be daunting and ad hoc, being dependent on a biologist's area of expertise.

(iii) Single-gene analysis may miss important effects on pathways. Cellular processes often affect sets of genes acting in concert. An increase of 20% in all genes encoding members of a metabolic pathway may dramatically alter the flux through the pathway and may be more important than a 20-fold increase in a single gene.

(iv) When different groups study the same biological system, the list of statistically significant genes from the two studies may show distressingly little overlap (3)"

To be clear, I am not necessarily recommending that the authors use GSEA specifically, but some sort of "second generation"/"Functional Class Scoring" pathway analysis (including GSEA or recent improvements on it) is probably the better approach to use than simple differential expression and averaging. Please take a look at the following review by Butte et al for reference: Khatri P, Sirota M, Butte AJ (2012) Ten Years of Pathway Analysis: Current Approaches and Outstanding Challenges. PLOS Computational Biology 8(2): e1002375. https://doi.org/10.1371/journal.pcbi.1002375

Response:

Authors thank R3 for proposing the use of functional class scoring analyses, such as gene-set enrichment analysis (GSEA), to test the robustness of our outcomes. Along this suggestion, we have analysed expression data from cohort A using GSEA4.1 software (Subramanian et al, Proc Natl Acad Sci, 2005). These additional analyses, summarized in **Figure 5** below, verified our analyses of pre-defined gene-sets, differential gene-expression and ingenuity pathway analysis.

Along R3's suggestion we have added GSEA to our revised flow of analyses, which now comprises the following steps in the identification and validation of T cell evasive mechanisms that underly spatial immunophenotypes. The first step, which was aimed at generating hypotheses regarding T

cell evasive mechanisms that underly different spatial immunophenotypes, constituted a biased approach using gene-sets related to T cell evasion. This approach relied on averaging expressions of unidirectional gene-set (for details on used gene sets, see Hammerl at al., CCR, 2019). The second step constituted an unbiased approach, where we used differential gene expression followed by ingenuity pathway analysis, which showed high concordance with our first step. In a third step, we exploited multiplex-IF stainings to validate and extract spatial information regarding T cell evasive mechanisms identified via the first 2 steps. The fourth and last step now covers GSEA, an unbiased, next generation analysis tool, which again confirmed our main findings: the excluded phenotype being characterized by glycolysis and collagen-10 deposition and association with TGFβ signaling; the ignored phenotype characterized by the presence of myeloid cells and association with WNT signaling; and the inflamed phenotype characterized by T cell co-inhibition and association with necrosis. See **Figures 4, 5, and Supplementary Figure 9** (all in revised manuscript) for details.

In the revised manuscript, we have included GSEA in the Materials and Methods (see **line#: 663-665**) and Results section (see **line#: 240-259**) and added below **Figure 5** as **Supplementary Figure 10**.

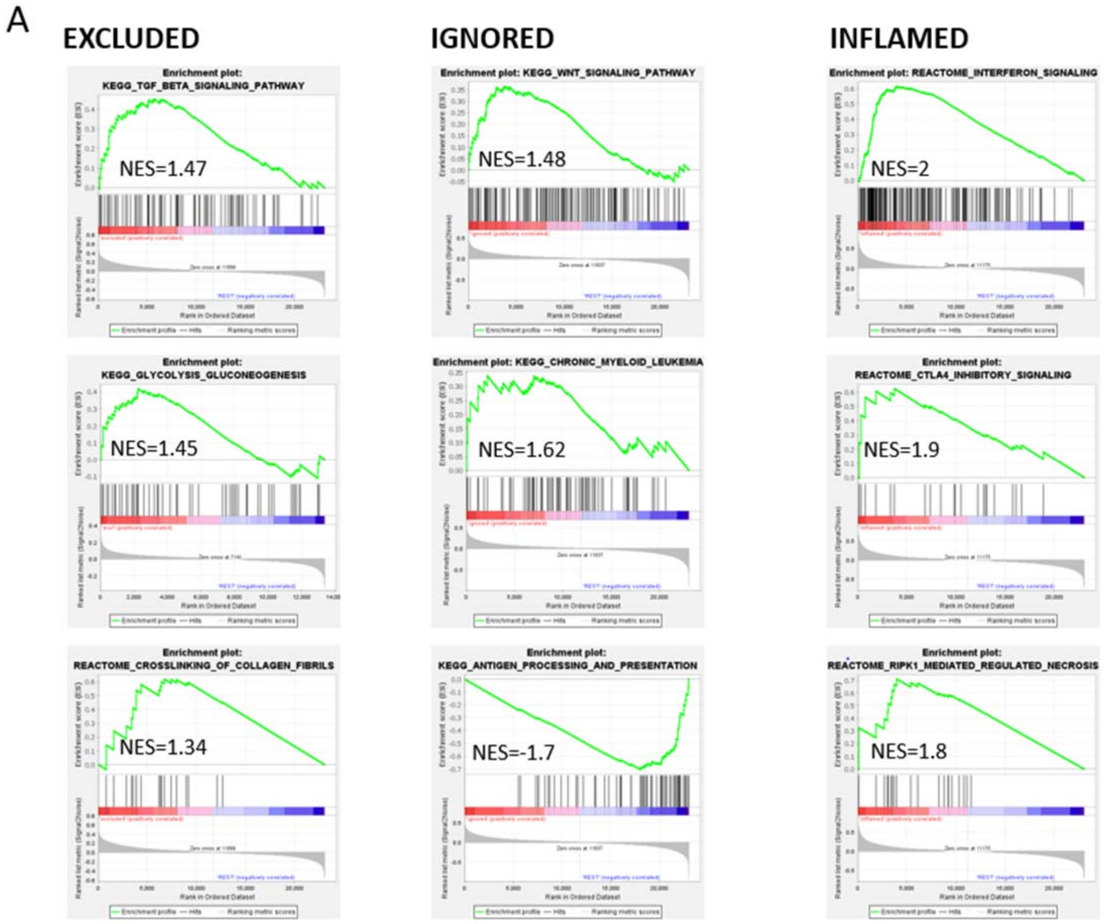

B

Excluded

KEGG	NES	REACTOME	NES
TGF_BETA_SIGNALING_PATHWAY	1.478134	CRMPS_IN_SEMA3A_SIGNALING	1.6600893
MELANOMA	1.400128	FGFR2_MUTANT_RECEPTOR_ACTIVATION	1.6470528
TASTE_TRANSDUCTION	1.398243	FRS_MEDIATED_FGFR2_SIGNALING	1.6412201

Ignored

KEGG	NES	REACTOME	NES
STEROID_BIOSYNTHESIS	1.703354	MAPK_TARGETS_NUCLEAR_EVENTS	1.7724794
VASOPRESSIN_REGULATED_WATER_REABSORPTION	1.688975	MET_PROMOTES_CELL_MOTILITY	1.7694103
FOCAL_ADHESION	1.633313	SYNAPTIC_ADHESION_LIKE_MOLECULES	1.7659823

Inflamed

KEGG	NES	REACTOME	NES
ALLOGRAFT_REJECTION	1.82267	TNF_SIGNALING	2.068332
AUTOIMMUNE_THYROID_DISEASE	1.835758	REGULATION_OF_IFNA_SIGNALING	2.0640402
GRAFT_VERSUS_HOST_DISEASE	1.838929	REGULATION_OF_TNFR1_SIGNALING	2.053227

Figure 5. Gene-set enrichment analysis for spatial immunophenotypes in TNBC. A. Enrichment plots from KEGG and REACTOME databases showing those gene-sets and pathways that are specifically enriched in the excluded (left panel), ignored (middle panel) and inflamed (right panel) phenotypes in TNBC which have also been identified using DE and IPA analysis (see **Figure 4**). **B**. Top 3 enriched pathways according to KEGG and REACTOME databases with normalized enrichment scores (NES) per spatial immunophenotype.

MINOR COMMENTS:

1: *Figure 2F is inadequately explained in the figure legend of the corresponding text. What is the predictor here? Do the various panels show predictions for ignored-vs-others, excluded-vs-others, and ignored/excluded-vs-inflamed in predicting response to ICI? I recommend clarifying the text here.*

<u>Response:</u> Authors thank R3 for pointing to lack of clarity regarding the legend to Figure 2F. We have amended the figure legend accordingly (see **line# 458-462**).

2 and 3: *A brief discussion on the limitations of visual assessment (ambiguity, non-representativeness of examined regions compared to full tumor, etc) is needed. The authors should explain in the limitations that future studies, wherever practically possible, can use quantitative computational approaches instead for this task. Likewise, a brief discussion of the limitations of digital image analysis is needed. For example, the authors state in the methods that "Tissue-segmentation, cell-segmentation and phenotyping of individual cells was performed using Inform software". While commercial software tends to do a reasonable job for many tasks, it should be noted that misclassifications and errors in segmentation are not uncommon. The authors should mention this somewhere in the limitations as a confounder to the analysis.*

<u>Response:</u> We agree that multiplex immunofluorescence with digital image analysis of defined regions may not fully reflect tumor heterogeneity, and computed assignments of tissue compartments and immune cells may harbor a certain degree of misclassification.
In line with R3's recommendations, we have amended text regarding quantitative computational approaches as well as limitations of digital analysis in the Discussion section of the revised manuscript (**see line# 419-422**).

Reviewers' Comments:

Reviewer #1:
Remarks to the Author:

The authors have substantially and sufficiently revised the content and clarity of the manuscript with appropriate inclusion of new tables, figures and expanded methodological details that directly address the reviewer's major concerns. It would be prudent, however, for the authors to review the methodological citations so as to prevent omissions, an example being lack of citation for the Combat batch correction method.

Reviewer #2:
None

Reviewer #3:
None
